# METTL3 depletion blocks vesicular stomatitis virus replication in pancreatic cancer cells through the establishment of an intrinsic antiviral state

**DOI:** 10.1128/jvi.02284-24

**Published:** 2025-04-11

**Authors:** Cassandra Catacalos-Goad, Jacob Hawkins, Quinton Krueger, Nathaniel Foret, Valery Z. Grdzelishvili

**Affiliations:** 1 Department of Biological Sciences, University of North Carolina at Charlotte 14727, Charlotte, North Carolina, USA; 2 Computational Intelligence to Predict Health and Environmental Risks (CIPHER) Center, University of North Carolina at Charlotte 14727, Charlotte, North Carolina, USA; University Medical Center Freiburg, Freiburg, Germany

**Keywords:** vesicular stomatitis virus, METTL3, m6A RNA methylation, pancreatic ductal adenocarcinoma, pancreatic cancer, oncolytic, intrinsic antiviral state, interferon signaling, RIG-I, interferon lambda

## Abstract

Vesicular stomatitis virus (VSV) is a promising oncolytic virus (OV) against different malignancies, including pancreatic ductal adenocarcinoma (PDAC). In this study, we examined the role of methyltransferase-like 3 (METTL3), a catalytic subunit of the cellular writer complex that is responsible for N6-methyladenosine (m6A) RNA modification, as a potential host factor of VSV replication in PDAC cells. METTL3 was previously shown to be upregulated in PDAC, where it promotes cancer cell proliferation, invasion, and chemoresistance. The impact of METTL3 on life cycles of different viruses varies depending on both the virus and the cell type. Additionally, METTL3 plays a positive role in VSV replication in non-PDAC cells via m6A modification of VSV RNAs, which attenuates innate antiviral responses. In this study, we examined the role of METTL3 in 10 different human PDAC cell lines and uncovered two distinct outcomes. METTL3 depletion did not affect VSV replication in PDAC cell lines with defective innate antiviral signaling, suggesting that METTL3 is not directly involved in VSV replication. In contrast, METTL3 depletion dramatically inhibited VSV replication in PDAC cell lines with functional antiviral signaling. We show that this result is due to the RIG-I-dependent induction of a virus-independent, intrinsic antiviral state in METTL3-depleted PDAC cells. This intrinsic antiviral state was marked by type-III (but not type I or II) interferon secretion and constitutive overexpression of antiviral sensors [RIG-I (DDX58), MDA5 (IFIH1), and LGP2 (DHX58)], transactivators (STAT1, IRF7, and IRF9), and a diverse subset of antiviral effectors, including MX1, OAS1/2/3, and IFIT1/3.

**IMPORTANCE:**

Pancreatic cancer is a deadly and extremely challenging disease, making it essential to develop new treatment options and improve patient survival rates. One promising approach is the use of replication-competent “oncolytic viruses” designed to specifically target and destroy cancer cells while sparing healthy ones. To create effective oncolytic virus therapies for pancreatic cancer, it is crucial to identify host factors that influence the successful infection of cancer cells by these viruses. Here, we demonstrate that the cellular protein METTL3, which was previously shown to promote pancreatic cancer cell proliferation, invasion, and resistance to chemotherapy, plays a positive role in oncolytic virus replication in most of the tested human pancreatic cancer cell lines. We demonstrate that METTL3 depletion induces a chronic antiviral state that dramatically inhibits viral replication. Our study is important for understanding and improving oncolytic virus-based therapies.

## INTRODUCTION

Pancreatic ductal adenocarcinoma (PDAC) remains one of the most challenging malignancies, with a dismal prognosis largely due to late-stage diagnosis and limited therapeutic options (1, 2). Traditional treatments such as surgery, radiation, and chemotherapy often offer limited benefits, underscoring the urgent need for novel therapeutic strategies. One promising approach involves the use of oncolytic virus (OV) therapy, which employs replication-competent viruses to selectively infect and destroy cancer cells while sparing normal tissues (3–6).

Vesicular stomatitis virus (VSV) (order *Mononegavirales*, family *Rhabdoviridae*, subfamily *Alpharhabdovirinae*, genus *Vesiculovirus*) is a nonsegmented, negative-sense (NNS) RNA virus that exhibits natural oncolytic properties and can be genetically engineered to enhance its safety, “oncoselectivity” (preferential replication in cancer cells compared with nonmalignant cells), and efficacy against various tumors. VSV can induce direct oncolysis of infected cells, stimulate an antitumor immune response, and has the potential for combination with other therapies (7, 8). Importantly, VSV-based OVs are in phase I and II clinical trials (clinicaltrials.gov trials NCT01628640, NCT03120624, NCT04046445, NCT03865212, NCT03017820, NCT03647163, NCT06508463, NCT05644509, NCT03120624, NCT02923466, NCT03456908, NCT05846516). Alongside other OVs, VSV has potential as a future treatment for PDAC (9, 10). A better understanding of host factors affecting replication of VSV in PDAC cells to VSV and other OVs is critical to the development of rational OV approaches (10).

N6-methyladenosine (m6A) is an important RNA modification that significantly impacts various cellular processes, including RNA stability, splicing, translation, and decay (11–14). m6A has emerged as a key player in modulating viral infections, influencing both host cell and viral dynamics (15–18). Methyltransferase-like 3 (METTL3) protein is the core catalytic subunit of the m6A methyltransferase writer complex in human cells and most multicellular eukaryotes. This complex is composed of a METTL3 and METTL14 dimer (METTL3–METTL14) and several accessory proteins (19). The METTL3–METTL14 dimer acts as the catalytic subunit of the m6A writer, with METTL3 binding to the methyl donor S-adenosylmethionine (SAM), while METTL14 stabilizes the interaction between METTL3 and its RNA substrate for the METTL3-mediated methyl transfer to take place (19–21).

In PDAC, m6A is increasingly recognized for its role in mediating resistance to therapy (22) by affecting the stability and translation of various mRNAs that regulate critical functions such as cell survival, proliferation, and apoptosis (23–25). Furthermore, m6A plays a role in metastasis by regulating genes involved in epithelial-to-mesenchymal transition (EMT) and cell migration (26–28). Targeting METTL3 and other m6A regulators in PDAC currently presents a promising therapeutic strategy to address these resistance mechanisms and enhance the efficacy of existing treatments. STORM Therapeutics is currently in phase I clinical trials using a METTL3 inhibitor (STC-15) in patients with advanced malignancies (clinicaltrials.gov trial NCT05584111). Combinatorial approaches involving m6A-targeted therapies alongside other treatments are expected to be tested in the future to improve outcomes in PDAC patients. Therefore, understanding the impact of METTL3-targeting therapies on OV therapy is crucial.

The impact of METTL3 on life cycles of different viruses varies depending on both the virus and the cell type. Recently, several studies have shown that the level of m6A-enriched VSV RNA can affect the success of virus replication (29–33). Research in non-PDAC cells has shown that VSV replication was reduced and phosphorylation of IRF3, TBK1, and STAT1 was increased in RAW264.7, HeLa, and HEK293T cells when METTL3 was depleted, while METTL3 overexpression produced fewer interferons (IFNs) (29, 31). METTL3 is normally localized in the nucleus but was reported to directly interact with VSV RNA, mRNA, and antigenome in the cytoplasm of the infected cells (29). Together, these studies suggest that METTL3 depletion and consequent decrease in m6A RNA methylation of viral RNAs have an inhibitory effect on VSV replication via stimulation of antiviral signaling. However, there is no clear understanding of how altering host m6A levels remodels intracellular signaling before VSV infection.

In this study, we investigated METTL3 as a potential host factor of VSV replication in 10 different human PDAC cell lines and uncovered two distinct outcomes. METTL3 depletion did not affect VSV replication in PDAC cell lines with defective innate antiviral signaling, suggesting that METTL3 is not directly required for VSV replication. In contrast, METTL3 depletion inhibited VSV replication in PDAC cell lines with functional innate antiviral signaling. We demonstrate that this outcome arises from the RIG-I-dependent induction of a virus-independent, intrinsic antiviral state in METTL3-depleted PDAC cells. Our study shows an interesting interplay between METTL3, antiviral signaling, and VSV infection in PDAC, which is important for understanding how VSV’s oncolytic potential is regulated and offers valuable insights for OV therapy.

## MATERIALS AND METHODS

### Virus and cell lines

The recombinant VSV (Indiana serotype) virus VSV-ΔM51 (will be referred to as “VSV-ΔM51” in this study) was characterized previously (34), which lacks the methionine at amino acid position 51 of the matrix protein (M) and incorporates the GFP open reading frame (ORF) at position 5 within the viral genome, situated between the VSV G and L genes (34, 35). VSV-Mwt has the same GFP insertion as VSV-ΔM51 but a wild-type (wt) M gene (36). VSV-Mwt-P1 also has the wt M gene, but insertion of the GFP ORF at position 1 (P1) of the VSV genome results in virus attenuation and slower viral replication kinetics (34, 35). Baby hamster kidney fibroblast cells BHK-21 (ATCC CCL-10) were used to generate virus stock and to determine virus titer. All viral titers were determined by adding 5-fold serial dilutions of VSV-ΔM51 to BHK-21 cells in a 12-well plate. An agar overlay was used an hour after infection and at 24 hours (h) post-infection (p.i.) both fluorescent focus units per mL (FFU/mL) and plaque forming units per mL (PFU/mL) were calculated. To count FFUs, VSV-encoded GFP fluorescent foci were quantified using fluorescent microscopy. To quantify PFUs, cells were fixed and stained with crystal violet. The human PDAC cells used in this study were SUIT-2 (37), HPAF-II (38), AsPC-1 (39), Capan-1 (40), Capan-2 (41), CFPAC-1 (42), MIA PaCa-2 (43), HPAC (44), T3M4 (45), and HS766t (46) (Table 1). The immortal human pancreatic duct epithelial (HPDE) cell line (47) was also utilized in this study and cultured in Keratinocyte-SFM (Gibco). This cell line, created by introducing the E6 and E7 genes from human papillomavirus 16 into normal adult pancreatic epithelium, maintains a genotype similar to that of pancreatic duct epithelium and remains nontumorigenic in nude mice (47). Additionally, A549 (ATCC CCL-185), a human lung carcinoma cell line, was used in this study. The human origin of all tested PDAC cell lines was confirmed as previously described (48). SUIT-2, MIA PaCa-2, HS766t, HPAC, CFPAC-1, Capan-1, and A549 cells were maintained in Dulbecco’s modified Eagle’s medium (DMEM, Corning). Capan-2, AsPC-1, and T3M4 cells were maintained in Roswell Park Memorial Institute 1640 medium (Corning). HPAF-II and BHK-21 cells were maintained in Minimum Essential Medium (Corning). All cell growth media were supplemented with 10% fetal bovine serum (FBS, Gibco), 4 mM l-glutamine, 900 U/mL penicillin, 900 µg/mL streptomycin, and 1% nonessential amino acids. HPAF-II and BHK-21 cells were additionally supplemented with 17.5% glucose. All cells were kept in a 5% CO_2_ atmosphere at 37°C. For all experiments, cells were kept for no more than 15 passages. All described experiments were approved by the University of North Carolina at Charlotte Institutional Biosafety Committee (IBC).

**TABLE 1:** General information about human PDAC cell lines used in this study^*a*^

Cell line	Origin	Chemo or radiation therapy exposure?	Originally described
HPAF-II (ATCC: CRL-1997)	1982, metastasis (ascites), male, 44 years	N/A	(38)
AsPC-1 (ATCC: CRL-1682)	1982, metastasis (ascites), female, 62 years	Chemo and radiation	(39)
Capan-2 (ATCC: HTB-80)	1986, primary, male, 56 years	Chemo	(41)
CFPAC-1 (ATCC: CRL-1918)	1990, metastasis (liver), male, 26 years	N/A	(42)
MIA PaCa-2	1977, primary, male, 65 years	N/A	(43)
SUIT-2	1987, primary and metastasis (lymph node), male, 73 years	N/A	(37)
Capan-1 (ATCC: HTB-79)	1974, metastasis (liver), male, 40 years	Chemo	(40)
HPAC (ATCC: CRL-2119)	1985, primary, female, 64 years	N/A	(44)
T3M4	1978, metastasis (lymph node), male, 64 years	N/A	(45)
HS766t	1973, metastasis (lymph node), male, 64 years	N/A	(46)

^*a*^ Cell lines with “chemo” and/or “radiation” indicate treatments administered to the patient. N/A: information not available.

### Suppression of METTL3 expression using siRNA transfection

To inhibit the production of METTL3, cells were transfected with siRNAs targeting METTL3, RIG-I (DDX58), or cGAS (MB21D1) (Integrated DNA Technologies siRNA kits; Table 2). To target each gene, a pool of three siRNA sequences was used together. Negative control DsiRNA (“Scramble” siRNA sequences) were used as a control and were provided by the manufacturer in the same siRNA kit (Integrated DNA Technologies, Cat. No. 51-01-14-04). Cells were plated at 0.14 × 10^6^ cells per well in a 12-well plate for 35% confluence and incubated at 5% CO_2_ at 37°C for 24 h before transfection. For cell transfection, siRNA and transfection reagent (Invitrogen RNAi Max Lipofectamine, Cat. No. 13778150) were suspended in supplement and antibiotic-free medium (Opti-MEM, Invitrogen) and mixed 1:1 by volume before being added to cells. 24 h post-transfection, 0.5 mL of supplement and antibiotic-free medium (Opti-MEM, Invitrogen) was added to cells. Cell collection or viral infection took place 48 h post-transfection for optimal knockdown.

**TABLE 2:** siRNA sequences used in this study

siRNA name	siRNA sequence
Ri.METTL3.13.1-SEQ1	CUAAACCUGAAGAGUGAUAUUUGTA
hs.Ri.METTL3.13.1-SEQ2	UACAAAUAUCACUCUUCAGGUUUAGCU
hs.Ri.METTL3.13.2-SEQ1	GGAUACCUGCAAGUAUGUUCACUAT
hs.Ri.METTL3.13.2-SEQ2	AUAGUGAACAUACUUGCAGGUAUCCAU
hs.Ri.METTL3.13.3-SEQ1	AAUCUAUGGCAUGAUUGAAAGACTA
hs.Ri.METTL3.13.3-SEQ2	UAGUCUUUCAAUCAUGCCAUAGAUUUC
hs.Ri.DDX58.13.1-SEQ1	CAGAAUCUUAGUGAGAAUUCAUGTC
hs.Ri.DDX58.13.1-SEQ2	GACAUGAAUUCUCACUAAGAUUCUGGC
hs.Ri.DDX58.13.2-SEQ1	AUAUCAGGUCCUCAAUCUUCAGCTA
hs.Ri.DDX58.13.2-SEQ2	UAGCUGAAGAUUGAGGACCUGAUAUCA
hs.Ri.DDX58.13.3-SEQ1	GUAGUAUUCUUACUAAGACCCAATA
hs.Ri.DDX58.13.3-SEQ2	UAUUGGGUCUUAGUAAGAAUACUACUA
hs.Ri.MB21D1.13.1-SEQ1	GCAACUUAAUUGACAAAAGAAGUAA
hs.Ri.MB21D1.13.1-SEQ2	UUACUUCUUUUGUCAAUUAAGUUGCUA
hs.Ri.MB21D1.13.2-SEQ1	AAGGUGAAAUAUUAUCAGCUUCUAA
hs.Ri.MB21D1.13.2-SEQ2	UUAGAAGCUGAUAAUAUUUCACCUUCU
hs.Ri.MB21D1.13.3-SEQ1	AGAACUAGAGUCACCCUAAAUCCTG
hs.Ri.MB21D1.13.3-SEQ2	CAGGAUUUAGGGUGACUCUAGUUCUUA

### METTL3 inhibition using STM2457

STM2457 (Selleck Chem, Cat. No. S9870) is a selective inhibitor of METTL3 catalytic activity. In a clear, 96-well plate, cells (1.2 × 10^4^) were seeded to reach ~35% confluence the following day. STM2457 or DMSO was suspended in DMEM media (0.1% DMSO in DMEM) (Corning) and added to cells for 48 h before or/and 1 h after viral infection took place. Virus-encoded GFP fluorescence was monitored at various time points over 144 h p.i. using a fluorescence multi-well plate reader, with GFP fluorescence readings taken at 485/530 nm on the Tecan Infinite F200.

### Virus replication kinetics

Cells were plated onto 96-well plates. Virus dilutions were prepared in serum-free DMEM. After washing cells once with PBS, VSV-ΔM51 was added and allowed to infect for 1 h at 37°C. Subsequently, the virus-containing medium was removed, and fresh DMEM containing 5% FBS was replenished, followed by further incubation at 37°C in 5% CO_2_ for the duration of the experiment. Virus-encoded GFP fluorescence was monitored at various time points over 120 h using a fluorescence multi-well plate reader, with GFP fluorescence readings taken at 485/530 nm on the Tecan Infinite F200.

### Cell viability and cell cytotoxicity assays

Cells were seeded in a 96-well plate layout and were either VSV-ΔM51-infected or mock-infected (as a negative control). For cell viability, WST-8 (Dojindo, CK04) was added to each well for 4 h at 37°C in 5% CO_2_, then read at 450 nm using a multi-well plate reader. For cell cytotoxicity, CytoTox-Glo reagent (Promega, G9290) was added to each well for 15 min at room temperature, then luminescence was measured. Alternatively, the crystal violet (CV) cell cytotoxicity assay was used. Cells were VSV-ΔM51-infected at a MOI of 0.1 (based on VSV-ΔM51 titer on BHK-21) or mock-infected for 72 h after a 48 h transfection with either siSCR or siMETTL3. To determine VSV-ΔM51-mediated cytotoxicity, we stained cells with crystal violet.

### Western blot analysis

Cells were seeded into 12-well plates to reach ~35% confluence. After 24 h, cells were transfected with METTL3 or scramble siRNA as previously explained. 48 h post-transfection, the medium was removed, and cells were washed once with PBS. Virus was then added at MOI of 0.1 in serum-free DMEM medium and incubated for 1 h at 37°C. After 1 h of incubation, the medium was removed, and 5% FBS-containing medium was added to the cells. Cells were then lysed, and total protein was isolated 24 h p.i. using a buffer containing 1 M Tris-HCl (pH 6.8), 10% glycerol, 2% SDS, 5% beta-mercaptoethanol, and 0.02% (wt/vol) bromophenol blue. Total protein was separated by electrophoresis on 10% or 15% SDS-PAGE gels and electroblotted onto polyvinyl difluoride (PVDF) membranes. Membranes were blocked by using 5% nonfat powdered milk or BSA in TBS-T [0.5 M NaCl, 20 mM Tris (pH 7.5), 0.1% Tween 20] for 1 h at room temperature. Membranes were then incubated in TBS-T with 5% BSA or milk with 0.02% sodium azide and a 1:5,000 dilution of rabbit polyclonal anti-VSV antibodies (raised against VSV virions), a 1:1,000 dilution of rabbit Phospho-Stat1 (Tyr701) (Cell Signaling, Cat. No. 9167S), a 1:1,000 dilution of rabbit anti-STAT1 (Cell Signaling, Cat. No. 14994T), a 1:500 dilution of rabbit anti-METTL3 (ThermoFisher Scientific, Cat. No. 15073-1-AP), a 1:1,000 dilution of rabbit anti-RIG-I (Cell Signaling, 4200S), a 1:1,000 dilution of rabbit anti-cGAS (Cell signaling, 15102S), or a 1:1,000 dilution of rabbit anti-MX1 (Proteintech, Cat. No. 13750-1-AP). Starbright Blue 700 goat anti-rabbit IgG fluorescent secondary antibodies (Bio-Rad, Cat. No. 12004161) at 1:5,000 dilutions were used for fluorescent western blotting detection using the ChemiDoc MP imaging system from Bio-Rad. To detect total protein, the membranes were stained with Coomassie Blue stain.

### Interferon sensitivity assay

Cells were seeded into 96-well plates to reach either ~30% or ~80% confluence and were given 24 h to adhere. Cells were then treated with either human IFN-α (Calbiochem, 407294), IFN-λ1 (R&D Systems 1598-IL-025), IFN-λ2 (R&D Systems 1587-IL-025), or IFN-λ3 (R&D Systems 5259-IL-025) [at 100 ng/mL, 10 ng/mL, 1 ng/mL, 0.1 ng/mL, or 0 ng/mL (virus only with 0.1% DMSO)] for 24 h (~80% confluent cells) or 48 h (~30% confluent cells). Next, the medium was removed, and cells were washed once with PBS. The virus was then added at MOI of 0.05 (based on BHK-21 titer) in a serum-free medium and incubated for 1 h at 37°C. After 1 h incubation, the medium was removed, and fresh medium (5% FBS) was added. Virus-encoded GFP fluorescence was measured at 485/535 nm periodically over a 120 h time course on the Tecan Infinite F200.

### Conditioned media assay

SUIT-2 cells were seeded for ~35% confluency the following day. Either scramble or METTL3 siRNA was transfected into cells. After 48 h post-transfection, medium was collected. SUIT-2 cells were then seeded at ~80% confluency, and the medium (from SUIT-2 transfected cells) was added to SUIT-2 cells 24 h before infection with VSV-ΔM51 at MOI of 0.05 (based on VSV-ΔM51 titer on BHK-21) or mock treated. Virus-encoded GFP fluorescence was measured at 485/535 nm periodically over a 120 h time course on the Tecan Infinite F200.

### Cytokine array

Cells were seeded into 12-well plates to reach 35% confluence and were incubated for 24 h to adhere. Cells were then transfected with METTL3 or scramble siRNA as previously explained. After 48 h, cell supernatants (for cytokine array), RNA (for RNA-seq), and lysates (for western blot) were collected and stored at −80°C. Supernatants were then sent to EVE Technologies for the Human Interferon 9-Plex Discovery Assay Array (Eve Technologies, Cat. No. HDIFN9).

### RNA isolation, RNA-seq analysis, and gene expression analysis

Three biological replicates were used for each treatment condition for RNA-seq. SUIT-2 and MIA PaCa-2 cell lines were seeded into 12-well plates (0.14 × 10^6^ cells per well) to reach ~35% confluence in DMEM containing 10% FBS. Cells were given 24 h to adhere. Cells were then transfected with either METTL3 or scramble siRNA as previously described. After 48 h, cellular RNA was isolated with TRIzol (Life Technologies) according to the manufacturer’s protocol. The RNA samples were then sent to GeneWiz for RNA-seq analysis. Sequence reads were trimmed to remove adapter sequences and low-quality bases using Trimmomatic v.0.36. The trimmed reads were mapped to the *Homo sapiens* GRCh38 reference genome available on ENSEMBL using the STAR aligner v.2.5.2b. Unique gene hit counts were calculated by using featureCounts from the Subread package v.1.5.2. The hit counts were summarized and reported using the gene_ID feature in the GRCh38 annotation file. Only unique reads that fell within exon regions were counted. After the extraction of gene hit counts, the gene hit counts table was used for downstream differential expression analysis (see “Data Availability Statement”). Using DESeq2, pairwise comparisons were performed. The Wald test was used to generate *P*-values and log2 fold changes. Genes with an adjusted *P*-value < 0.05 and absolute log2 fold change >1 were identified as differentially expressed.

### Statistical analysis

All statistical analyses (except for the RNA-seq analysis that is described above) were performed using GraphPad Prism 9.3.1 software. Tests used are indicated in the legends of the figures.

## RESULTS

### Two outcomes of METTL3 depletion on VSV-ΔM51 replication in various human PDAC cell lines

**Fig 1:**
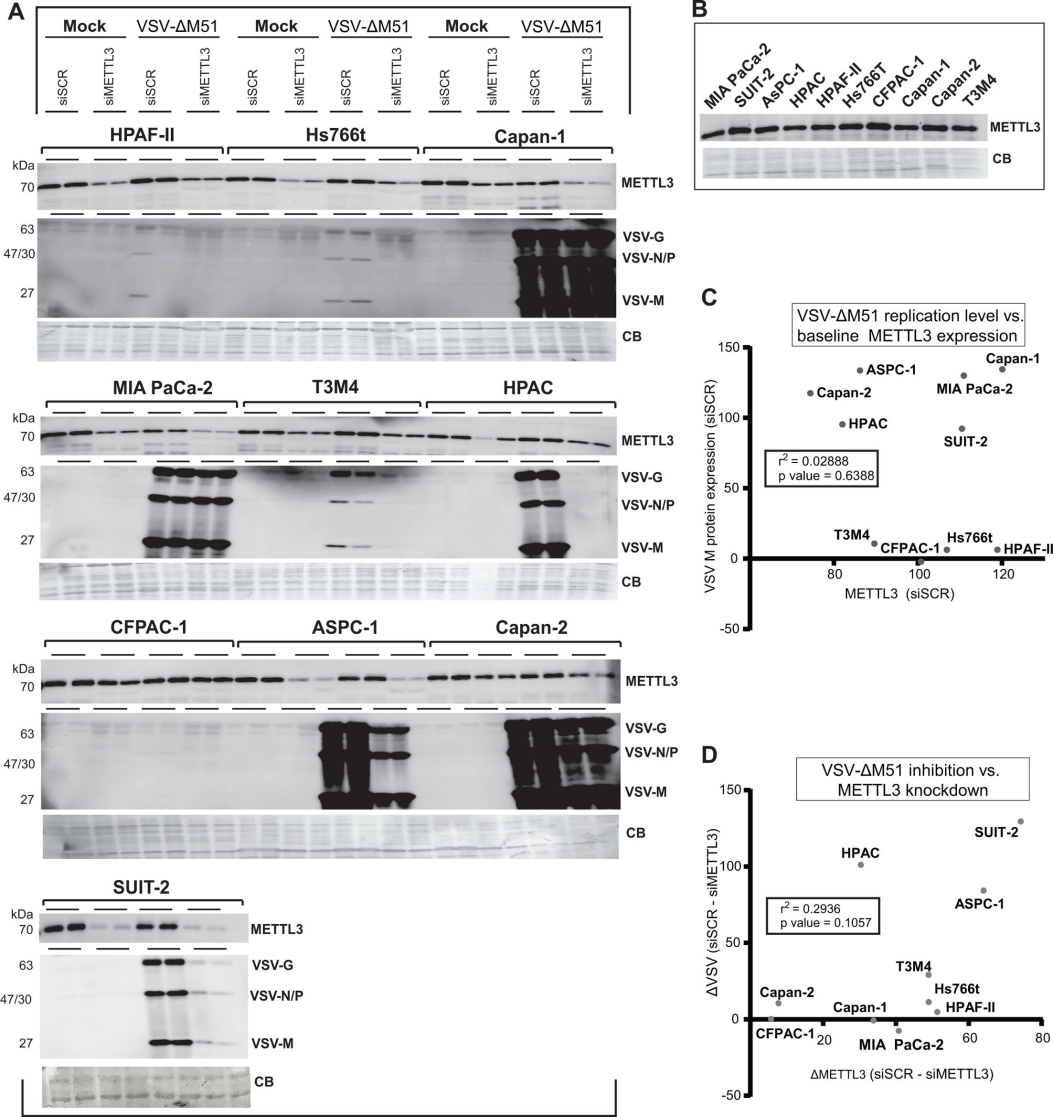
METTL3 and VSV protein expression in 10 PDAC cell lines. (**A**) Effect of siRNA-mediated METTL3 downregulation on replication of VSV-ΔM51 in 10 different human PDAC cell lines. Cells were either transfected with siSCR or siMETTL3 (25 nM) for 48 h before either VSV-ΔM51 or mock infection for 24 h. Cells were infected with VSV-ΔM51 at an MOI 1 (CFPAC-1, HPAF-II, and Hs766t), 0.1 (HPAC, SUIT-2, and T3M4), or 0.01 (ASPC-1, Capan-1, Capan-2, and MIA PaCa-2). The MOI was based on VSV-ΔM51 titer on BHK-21. Protein samples were analyzed by western blotting. Equal protein loading was verified by Coomassie Blue (CB) staining of the membranes. (**B**) Relative levels of METTL3 in uninfected cell lines. (**C**) Correlation between VSV-ΔM51 replication and baseline METTL3 expression. ImageJ software was used to quantify protein levels of VSV-M (siSCR + VSV-ΔM51) and METTL3 (siSCR + mock). (**D**) Correlation between VSV-ΔM51 inhibition and METTL3 knockdown. ImageJ software was used to quantify protein levels of VSV-M and METTL3 in both (siSCR + VSV-ΔM51) and (siMETTL3 + VSV-ΔM51) conditions normalized by a nonspecific band on each gel representing relative protein loading for each sample. Protein levels of siMETTL3 were subtracted from siSCR to determine the degree of knockdown (ΔMETTL3) and the degree of VSV-ΔM51 replication (ΔVSV). (**C, D**) The correlation coefficient was then generated based on this quantification described in C and D.

Our previous analyses of a large set of human PDAC cell lines have shown they are highly heterogeneous in their gene expression and phenotypic characteristics, including their permissiveness to VSV and other OVs (7–10, 48–50). Consequently, we chose to investigate the relationship between basal METTL3 expression levels and VSV replication, as well as the effects of METTL3 depletion on VSV replication across a panel of 10 human PDAC cell lines (Table 1). This panel included cell lines that were previously designated in our studies as “super resistant” (CFPAC-1, HPAF-II, and Hs766t), moderately permissive (HPAC, SUIT-2, and T3M4), or highly permissive (AsPC-1, Capan-1, Capan-2, and MIA PaCa-2) (9, 48, 49). This study focused on a widely used oncolytic recombinant VSV-ΔM51, which has a deletion of the methionine amino acid at position 51 in the VSV matrix (VSV-M) protein. This mutation improves VSV-ΔM51 oncoselectivity by attenuating VSV-ΔM51 in normal cells by preventing the VSV-M from blocking nucleocytoplasmic transport of cellular mRNA, including transcripts associated with antiviral responses (51–53). Additionally, a green fluorescent protein (GFP) reporter gene is inserted within the VSV-ΔM51 genome between the VSV G and L genes. To examine the relationship between basal METTL3 expression levels and VSV protein accumulation, as well as the effects of METTL3 depletion on VSV protein accumulation, PDAC cells were either treated with scramble siRNA (siSCR) or METTL3 siRNA (siMETTL3) for 48 h before being infected with VSV-ΔM51 at a multiplicity of infection (MOI) of 1 (CFPAC-1, HPAF-II, and Hs766t), 0.1 (HPAC, SUIT-2, and T3M4), 0.01 (AsPC-1, Capan-1, Capan-2, and MIA PaCa-2), or mock-infected (underwent the same treatment as VSV-ΔM51 infection but with no virus) (Fig. 1A). In addition, METTL3 levels were examined in untreated PDAC cell lines (Fig. 1B). For infections, the MOI was calculated in all experiments in this study based on VSV-ΔM51 titer on BHK-21, a highly permissive baby hamster kidney cell line widely used for VSV-ΔM51 amplification. The MOI 0.01–1 range was used based on our previous studies to normalize the levels of initial infection in different cell lines (9, 48, 49). To examine the accumulation of METTL3 and viral proteins, total protein was isolated at 24 hp.i., and METTL3 and VSV-encoded proteins (G, N, P, and M) were analyzed by western blotting (the effect on METTL3 depletion on *de novo* virion production will be discussed later in this manuscript). First, in agreement with our previous studies, we observed dramatic differences in VSV protein accumulation between siSCR-treated VSV-ΔM51-infected PDAC cell lines. The lowest level of viral protein accumulation was observed in HPAF-II, Hs766t, and CFPAC-1, intermediate levels were observed in T3M4, SUIT-2, and HPAC, and the highest in Capan-1, MIA PaCa-2, AsPC-1, and Capan-2. The levels of VSV protein accumulation did not correlate with basal levels of METTL3 expression in siSCR-treated cells (Fig. 1A and C) or untreated cells (Fig. 1B). Regarding the effect of METTL3 downregulation on VSV protein accumulation, no increase in viral protein accumulation was observed in METTL3-downregulated cells in any of the cell lines. Instead, two distinct outcomes were noted. In most cell lines, we observed clear inhibition of virus replication, ranging from dramatic inhibition in T3M4, HPAC, and SUIT-2 cells to somewhat weaker negative effects in AsPC-1 and Capan-2 cells. VSV-ΔM51 inhibition was also observed in HPAF-II, Hs766t, and CFPAC-1 cell lines, although the basal levels of VSV protein accumulation in those cell lines were very low (Fig. 1A). In contrast to this first outcome (inhibition of VSV-ΔM51 replication after METTL3 downregulation), we observed no effect of METTL3 downregulation on VSV-ΔM51 replication in Capan-1 and MIA PaCa-2 cell lines. As certain PDAC cell lines were more successful with siRNA-mediated METTL3 knockdown (e.g., CFPAC-1 was unsuccessful), we examined for a possible correlation between the efficiency of METTL3 downregulation and VSV-ΔM51 inhibition. As shown in Fig. 1D, no correlation was found, suggesting that the observed lack of effect of METTL3 depletion on VSV protein accumulation in some cell lines (e.g., MIA PaCa-2) was not due to an insufficient METTL3 inhibition. To explore the phenotypic differences between PDAC cell lines where METTL3 depletion either dramatically inhibited or had no impact on VSV protein accumulation, we selected the SUIT-2 and MIA PaCa-2 cell lines. As shown in Fig. 1C, both SUIT-2 and MIA PaCa-2 showed similar levels of basal METTL3 expression and VSV-ΔM51 replication. However, SUIT-2 exhibited the most significant negative impact of METTL3 downregulation on VSV-ΔM51 replication, while MIA PaCa-2 showed the least effect (Fig. 1D). These lines exhibited the most efficient knockdown within their respective groups (Fig. 1D) and were chosen for the subsequent stages of the study.

**Fig 2:**
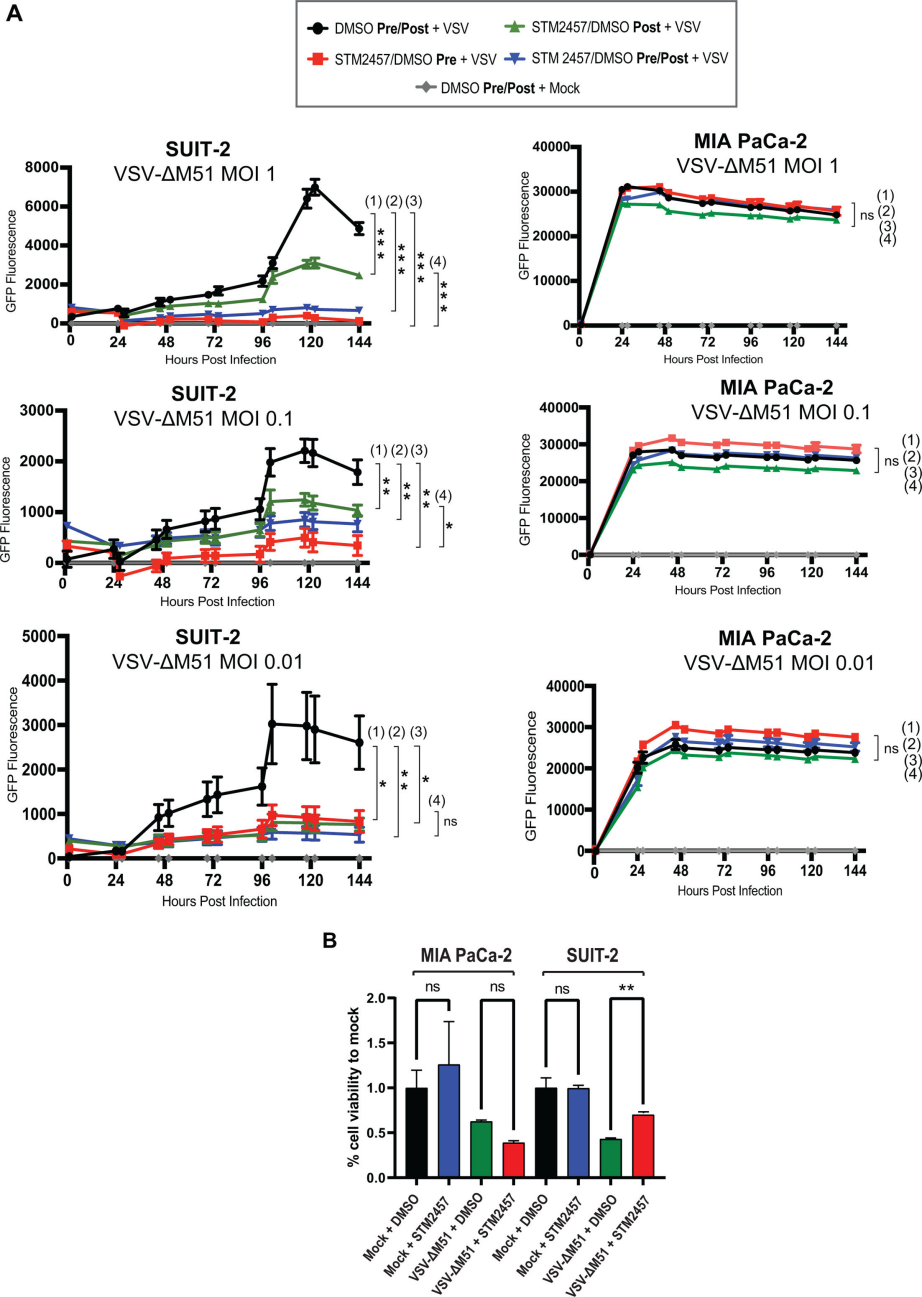
Effect of METTL3 inhibitor STM2457 on VSV-ΔM51 in SUIT-2 and MIA PaCa-2. (**A**) Cells were treated with STM2457 (10 mM) with 0.1% DMSO or control (0.1% DMSO only) either 48 h before (Pre), 1 h after (Post), or 48 h before and 1 h after (Pre/Post) VSV infection at MOIs 1, 0.1, or 0.01 (based on VSV titer on BHK-21) or mock infection. GFP fluorescence was measured between 1 and 144 h p.i. Results were analyzed to determine significance using the Student’s *t*-test comparing at 144 h p.i. (192 h post-initial drug treatment): (1) 0.1% DMSO + VSV vs 10 mM STM2457 post-treatment + VSV (2); 0.1% DMSO + VSV vs 10 mM STM2457 pre/post-treatment + VSV (3); 0.1% DMSO + VSV vs 10 mM STM2457 pre-treatment + VSV (4); 10 mM STM2457 post-treatment +VSV vs 10 mM STM2457 pre-treatment + VSV. *P < 0.05, **P < 0.01, ***P < 0.001. ns, not significant. (**B**) Cell viability of STM2457 pre-treated only (or mock-pre-treated) and VSV-ΔM51-infected (or mock-infected) MIA PaCa-2 and SUIT-2 cells was measured at 120 h p.i. using a WST-8 cell viability assay. Each data point was divided by the average mock value to obtain the percentage viability to mock. The data points and error bars shown represent the means and standard deviation of the means, respectively. Results were analyzed to determine significance using a one-way ANOVA with Fisher’s least significant difference test between STM-2457 and DMSO control within mock- and VSV-ΔM51-treated samples. ns, not significant; **P  < 0.01.

**Fig 3:**
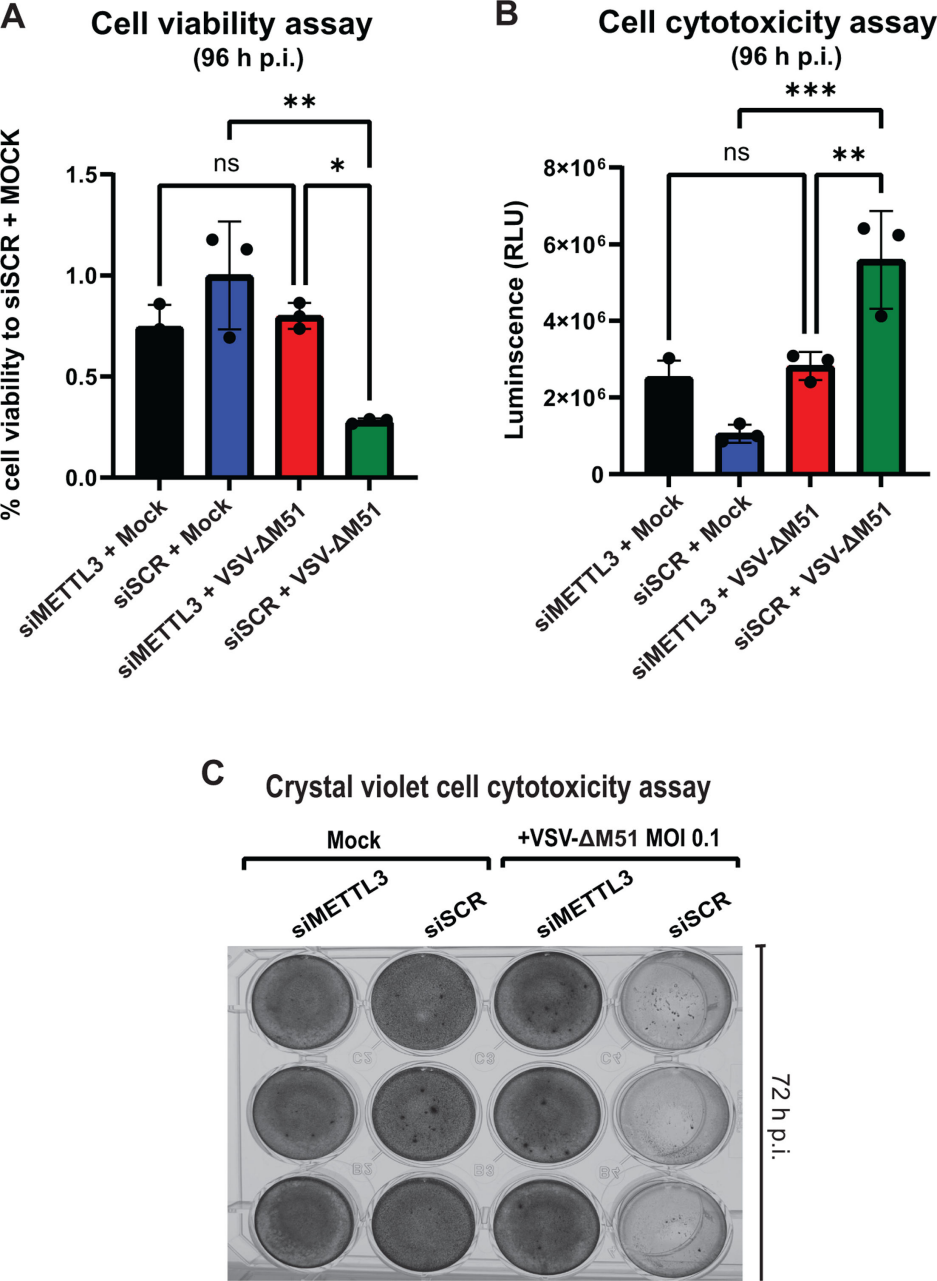
Effect of METTL3 depletion on cell viability and cell cytotoxicity. SUIT-2 cells were transfected with either siSCR or siMETTL3 48 h before VSV-ΔM51-infected or mock-infected for 96 h (**A and B**) or 72 h (**C**). (**A**) Cell viability was measured at 96 h p.i. using a WST-8 cell viability assay. (**B**) Cell cytotoxicity was measured at 96 h p.i. using a CytoTox-Glo Cytotoxicity Assay. The data points and error bars shown represent the means and standard deviation of the means, respectively. Results were analyzed to determine significance using a one-way ANOVA with Fisher’s least significant difference test between STM-2457 and DMSO control within mock- and VSV-ΔM51-treated samples. ns, not significant; *P  < 0.05. **P  < 0.01, ***P  < 0.001. (**C**) Crystal violet (CV) was used to stain SUIT-2 cells at 72 h p.i.

We next examined the impact of pharmacological inhibition of METTL3 activity on VSV-ΔM51 replication using STM2457, a selective, highly potent, S-adenosylmethionine (SAM) competitive inhibitor of METTL3 (54). We compared the effect of STM2457 on VSV-ΔM51 replication in SUIT-2 and MIA PaCa-2 cells when 10 μM of STM2457 in 0.1% DMSO or 0.1% DMSO alone was added 48 h pre-infection, 1 h post-, or pre- and post- (48 h/1 h) VSV-ΔM51 infection (Fig. 2A; “DMSO Pre/Post + mock” GFP was subtracted from each condition before plotting data, which in some cases resulted in negative GFP values). The treatment relative to infection was crucial to analyze as the effect of STM2457 is reversible; therefore, when removed from the medium, the cells may return to normal METTL3 activity. As shown in Fig. 2A, there was no significant inhibition of VSV-ΔM51 under any condition with STM2457 treatment with MIA PaCa-2. In SUIT-2 cells, while STM2457 showed VSV-ΔM51 inhibition under all tested conditions, the strongest inhibitory effect was observed when it was added pre-infection only, and there was no significant additive inhibitory effect of STM2457 when it was added both 48 h pre-infection and 1 h post-infection (Fig. 2A). To examine whether the different effects of STM2457 on VSV-ΔM51 replication were possibly due to toxic cell line-specific effects of this drug on cell viability, all tested groups were analyzed for cell viability 120 h p.i. using a WST-8 cell viability assay. As shown in Fig. 2B for uninfected (“Mock +DMSO” vs “Mock +STM2457”), STM2457 did not have any negative effect on cell viability in the absence of virus infection. While STM2457 had no negative impact on cell viability in infected MIA PaCa-2 that received STM2457 compared to control groups (Fig. 2B, MIA PaCa-2, “VSV-ΔM51 + DMSO” vs “VSV-ΔM51 + STM2457”), we observed statistically significant (*P*-value ≤ 0.05) increased cell viability in infected SUIT-2 cells that received STM2457 compared to control groups (Fig. 2B, SUIT-2, “VSV-ΔM51 + DMSO” vs “VSV-ΔM51 + STM2457”). This result demonstrated that METTL3 depletion had a protective effect in SUIT-2 cells (but not in MIA PaCa-2) against virus-mediated oncolysis, likely due to inhibition of VSV-ΔM51 replication. To examine if siRNA-based METTL3 depletion has a similar protective effect against virus-mediated cytotoxicity in SUIT-2 cells at 96 h p.i. with VSV-ΔM51 at a MOI of 0.1, we performed a cell viability (Fig. 3A) and cell cytotoxicity (Fig. 3B) assays. Although we observed a significant decrease in cell viability and increase in cell cytotoxicity between siSCR + Mock and siSCR + VSV-ΔM51, respectively, there was no significant difference in cell viability and cell cytotoxicity in our siMETTL3 transfected SUIT-2 cells after a 96 h infection with VSV-ΔM51 (Fig. 3A and B). In a separate experiment, we stained cells with crystal violet after infecting siMETTL3 or siSCR-transfected cells with VSV-ΔM51 at a MOI of 0.1. As shown in Fig. 3C, VSV-ΔM51 infection resulted in lysis of cancer cells in the mock (siSCR) treated cells 72 h p.i. However, siMETTL3 treatment completely suppressed VSV-ΔM51-mediated lysis of SUIT-2 cells (Fig. 3C). Together, the data presented in Fig. 3 demonstrated that METTL3 depletion protected cancer cells against virus-mediated oncolysis, likely due to inhibition of VSV-ΔM51 replication.

### METTL3 depletion inhibits VSV-ΔM51 replication via stimulation of antiviral innate immune signaling that does not require the presence of viral products

**Fig 4:**
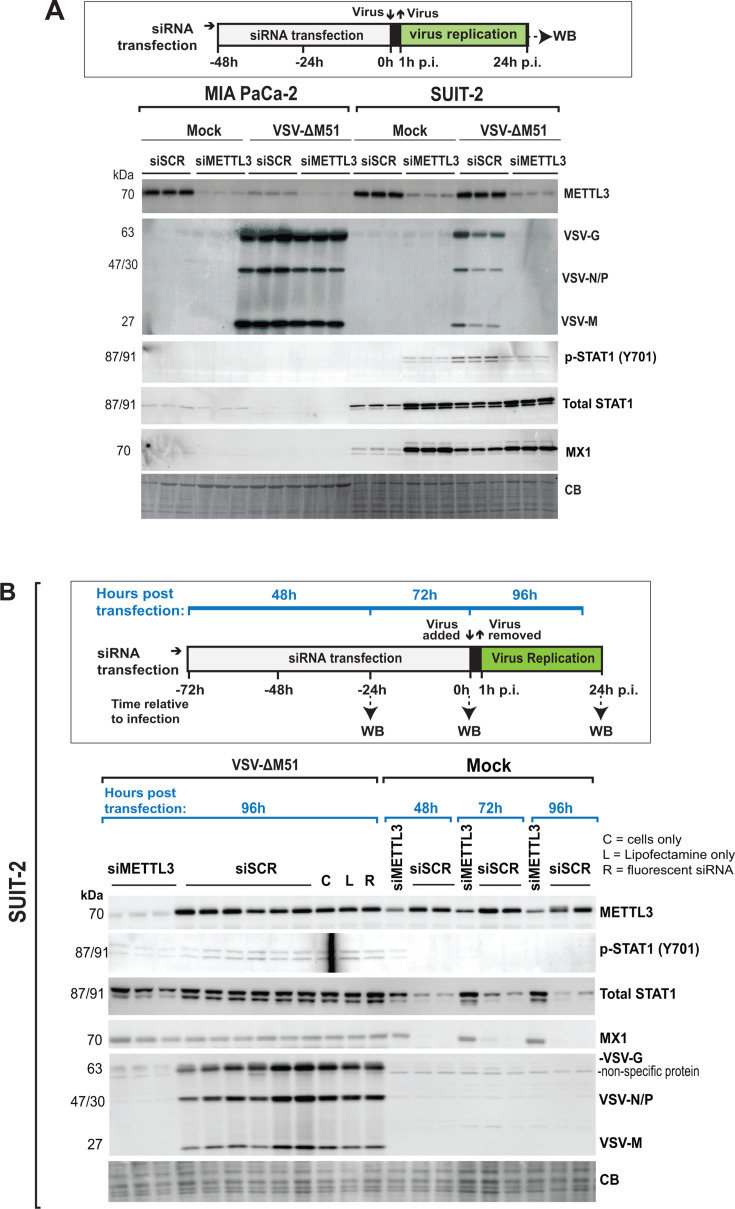
Effect of METTL3 depletion on antiviral status and VSV replication. (**A**) As illustrated in the schematic of the experiment, MIA PaCa-2 and SUIT-2 cells were transfected with either siSCR or siMETTL3 for 48 h and then infected with either mock or VSV (MOI 0.1; based on VSV titer on BHK-21) for 24 h. (**B**) As illustrated in the schematic of the experiment, protein samples were collected for SUIT-2 cells that were transfected with siSCR or siMETTL3 (no VSV infection) for 48 h, 72 h, or 96 h after, or for SUIT-2 cells that were transfected with siSCR or siMETTL3 for 72 h and then infected with VSV for 24 h p.i. (total time: 96 h post-transfection). Additional controls: Lipofectamine only (“L”; no siRNA), fluorescently labeled control siRNA (“R”), or neither lipofectamine nor siRNA (“C”; cells only). (**A**) and (**B**) Protein samples were analyzed by western blotting. Equal protein loading was verified by Coomassie Blue (CB) staining of the membranes.

The data in Fig. 1 and 2A show that depletion of METTL3 results either in inhibition (most PDAC cell lines) or no effect (MIA PaCa-2 and Capan-1) on VSV protein accumulation in different human PDAC cell lines. Interestingly, our previous studies showed that PDAC cell lines vary in their intrinsic and/or inducible innate antiviral status. While most PDAC cell lines have at least partially active antiviral responses, we showed that two highly VSV-permissive PDAC cell lines, MIA PaCa-2 and Capan-1, were most defective in their abilities to sense virus infection and mount an effective antiviral response (9, 48, 49). Intriguingly, the same two PDAC cell lines were also the only ones that showed no effect of METTL3 depletion on VSV-ΔM51 replication (Fig. 1A), and STM2457-mediated METTL3 inhibition had no statistically significant impact on VSV-ΔM51 replication in MIA PaCa-2 (Fig. 2A). Therefore, we hypothesized that METTL3 depletion affects VSV-ΔM51 replication via stimulation of antiviral signaling, which is at least partially functional in most PDAC cell lines, except for MIA PaCa-2 and Capan-1 (51–53). To test this hypothesis, we compared SUIT-2 and MIA PaCa-2 for their abilities to mount antiviral responses to VSV-ΔM51 and/or siMETTL3 (Fig. 4A). Both cell lines were treated with siSCR or siMETTL3 for 48 h and then infected with VSV-ΔM51 at MOI 0.1 or mock infected for 24 h. In agreement with Fig. 1A, siMETTL3 had a strong inhibitory effect on VSV-ΔM51 replication in SUIT-2 cells, but no effect in MIA PaCa-2 (Fig. 4A). Importantly, VSV-ΔM51 infection induced a strong antiviral response in SUIT-2 cells (“SUIT-2, mock-infection, siSCR” vs “SUIT-2, VSV-ΔM51, siSCR”), marked by upregulation of total STAT1, P-STAT1 (Y701), and MX1 (an important antiviral effector ISG, that was previously shown to inhibit VSV-ΔM51 replication) (48, 55–57), while no detectable response was observed in MIA PaCa-2 cells (“MIA PaCa-2, mock-infection, siSCR” vs “MIA PaCa-2, VSV-ΔM51, siSCR”). This result illustrates critical differences between SUIT-2 and MIA PaCa-2 in their abilities to mount an induced innate antiviral response to VSV-ΔM51 infection. Interestingly, a similar level of induction of total STAT1, P-STAT1 (Y701), and MX1 was observed even in uninfected SUIT-2 (but not in MIA PaCa-2) in response to siMETTL3 treatment (“SUIT-2, mock-infection, siSCR” vs “SUIT-2, mock-infection, siMETTL3”). The data in Fig. 4A support our hypothesis that METTL3 depletion affects VSV protein accumulation via stimulation of antiviral signaling and thus requires functional antiviral signaling (present in SUIT-2, but absent in MIA PaCa-2).

To further decipher the role of antiviral signaling in the impact of METTL3 depletion on VSV-ΔM51 replication in PDAC cells, we focused on SUIT-2. In our experiments described in Fig. 1A and 4A, cells were treated for 48 h with siMETTL3 or siSCR and then infected with VSV-ΔM51 for 24 h. Although we observed siMETTL3-mediated increased total STAT1, P-STAT1, and MX1 levels even in the absence of VSV-ΔM51 infection in mock-infected cells (“mock” in Fig. 4A) collected at 72 h post-transfection (48 h transfection plus 24 h infection), we wanted to examine if METTL3 depletion established the intrinsic antiviral state before cells were exposed to the virus (at 48 h post-transfection). To test this hypothesis, SUIT-2 cells were transfected with either siMETTL3 or siSCR for 48 h (this is when we would infect cells in Fig. 1A and 4A), 72 h (this is when we would collect cells for protein analysis in Fig. 1A and 4A), or 96 h, and looked at total STAT1, P-STAT1, and MX1 in the absence of VSV-ΔM51 infection (Fig. 4B). We also included in this experiment SUIT-2 cells transfected with siRNA for 72 h and then infected at an MOI of 0.1 for 24 h p.i. (Fig. 4B). In agreement with Fig. 1A and 4A, VSV protein accumulation was dramatically decreased in siMETTL3-treated cells compared with siSCR-treated cells. Importantly, in agreement with our hypothesis, total STAT1, P-STAT1, and MX1 were highly upregulated at 48 h after siRNA transfection at comparable levels with 72 h or 96 h post-siRNA transfection (Fig. 4B). Also, the level of total STAT1, P-STAT1, and MX1 in uninfected siMETTL3-treated cells was comparable to that with cells infected with VSV-ΔM51 with or without METTL3 depletion, suggesting that siMETTL3-induced antiviral state is activated at a similar level to the innate immune responses induced by VSV-ΔM51 infection. This experiment demonstrates that METTL3 depletion triggers an intrinsic antiviral state in cells before exposure to VSV-ΔM51. In the same experiment, we also examined the possibility that some nonspecific components of the siRNA treatment contained components that modulated induction of antiviral responses. To test this, SUIT-2 cells were treated with no Lipofectamine or siRNA (“C” in Fig. 4B), lipofectamine only (“L” in Fig. 4B), or lipofectamine with another nonspecific control siRNA (fluorescently labeled siRNA) provided by the manufacturer (“R” in Fig. 4B). There were no differences between P-STAT1, total STAT1, and MX1 when comparing “C”, “L”, “R”, or “siSCR” conditions, demonstrating that the observed impacts of siMETTL3 on VSV-ΔM51 replication were specific to METTL3 (Fig. 4B).

**Fig 5:**
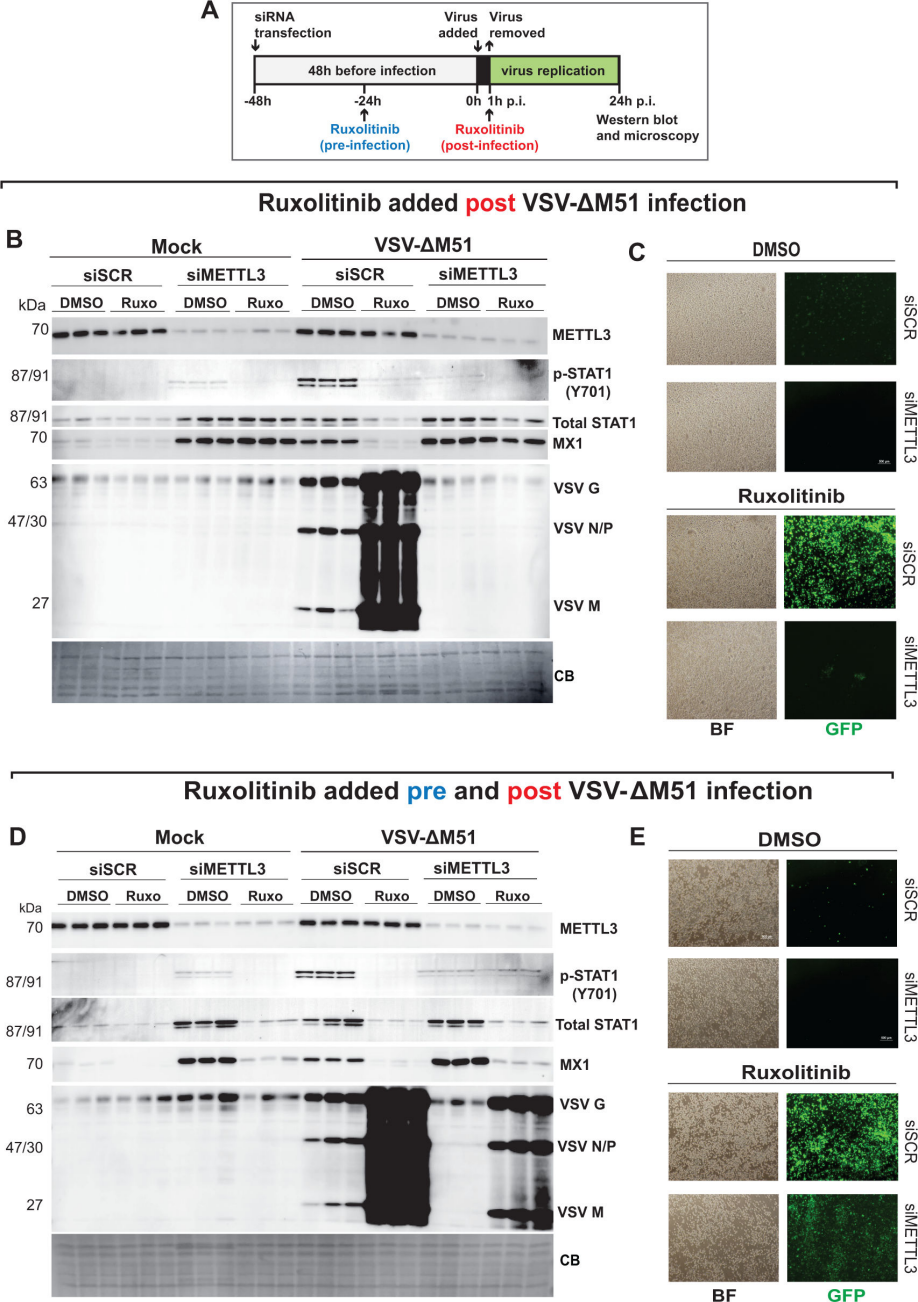
Effect of ruxolitinib on METTL3 depletion-mediated inhibition of VSV replication. (**A**) Schematic of experiments shown in B–E. SUIT-2 cells were either transfected with siSCR or siMETTL3 for 48 h and then infected with VSV at MOI 0.1 (based on VSV titer on BHK-21) for 24 h. Ruxolitinib was added either 1 h p.i. only or both 24 h before infection and 1 h p.i. (**B, C**) Ruxolitinib post-treatment only. SUIT-2 cells were treated with either ruxolitinib (0.5 μM) with 0.019% DMSO or control (0.019% DMSO only) at 1 h p.i. (**D, E**) Ruxolitinib pre-treatment and post-treatment. Cells were treated with either ruxolitinib (0.5 μM) with 0.019% DMSO or control (0.019% DMSO) 24 h after siRNA transfection (which was 24 h before infection with VSV). Then, 1 h p.i., the same amount of ruxolitinib (or DMSO control) was re-added to the cells. (**C, E**) Brightfield (BF) and GFP microscopy images were taken with Nikon camera at 24 h p.i. using 40× objective. (**B, D**) Protein samples were analyzed by western blotting. Equal protein loading was verified by Coomassie Blue (CB) staining of the membranes.

As we detected the increased total STAT1 and P-STAT1 levels in siMETTL3 SUIT-2 cells, we hypothesized the JAK/STAT pathway may be involved in the establishment of this antiviral state. To test this hypothesis, we used an FDA-approved highly specific JAK1/JAK2 inhibitor ruxolitinib (common brand name: Jakafi; indicated as “Ruxo” in Fig. 5) in SUIT-2 cells transfected with siSCR or siMETTL3 (Fig. 5A; 48 h siRNA treatment, followed by 1 h VSV-ΔM51 infection). We compared the impact of ruxolitinib added post-infection only (Fig. 5B and C) or pre- and post-infection (Fig. 5D and E) on siMETTL3-mediated reduction of VSV protein accumulation (Fig. 5B through E). Ruxolitinib is a reversible JAK1/JAK2 inhibitor; therefore, when ruxolitinib-containing medium is removed, its inhibitory effects diminish. For this reason, for both sets of experiments, the medium was replaced with ruxolitinib containing 0.1% DMSO vehicle 1 h p.i. (Fig. 5B through E), but for one set, ruxolitinib (or DMSO control) containing medium was also added 24 h pre-infection (Fig. 5D and E). We did not examine the ruxolitinib pre-infection only condition, as our previous studies showed that ruxolitinib pre-infection treatments are ineffective in PDAC cells due to the rapidly reversible nature of JAK1/JAK2 inhibition by this drug (58). Protein expression was analyzed using western blot (Fig. 5B and D) and VSV-ΔM51-driven GFP was analyzed using fluorescent microscopy (Fig. 5C and E) at 24 h p.i. Ruxolitinib strongly stimulated VSV-ΔM51 replication in siSCR-treated cells when added post (Fig. 5B and C) and pre + post (Fig. 5D and E) infection, confirming the critical role of the JAK/STAT pathway in antiviral responses of SUIT-2 cells to VSV-ΔM51 (7, 58). However, ruxolitinib could not suppress the effect of METTL3 depletion-mediated VSV-ΔM51 inhibition when it was added only post-infection (Fig. 5B and C), suggesting that cancer cells were in a fully active and irreversible intrinsic antiviral state before infection. Thus, the data show that 48 h pretreatment of cells with siMETTL3 was sufficient for the establishment of this antiviral state, despite inhibition of the JAK/STAT pathway through the addition of ruxolitinib after infection. In contrast, ruxolitinib had a dramatic suppressing effect on STAT1, P-STAT1, and MX1 upregulation when it was added also pre-infection, also resulting in a dramatic increase in VSV protein accumulation, ultimately partially reversing the phenotype of siMETTL3-transfected SUIT-2 (Fig. 5D and E). It is also important to note that VSV protein accumulation was greater in siSCR-treated SUIT-2 pre- and post-treated with ruxolitinib (Fig. 5B and C), compared to cells treated with ruxolitinib post-infection only (Fig. 5D and E), which is consistent with our previous study (58). In general, these data demonstrate that the establishment of the siMETTL3-mediated antiviral state is JAK/STAT dependent, it is established before virus infection, and this state could be overturned only when siMETTL3-treated cells were treated with ruxolitinib before infection.

**Fig 6:**
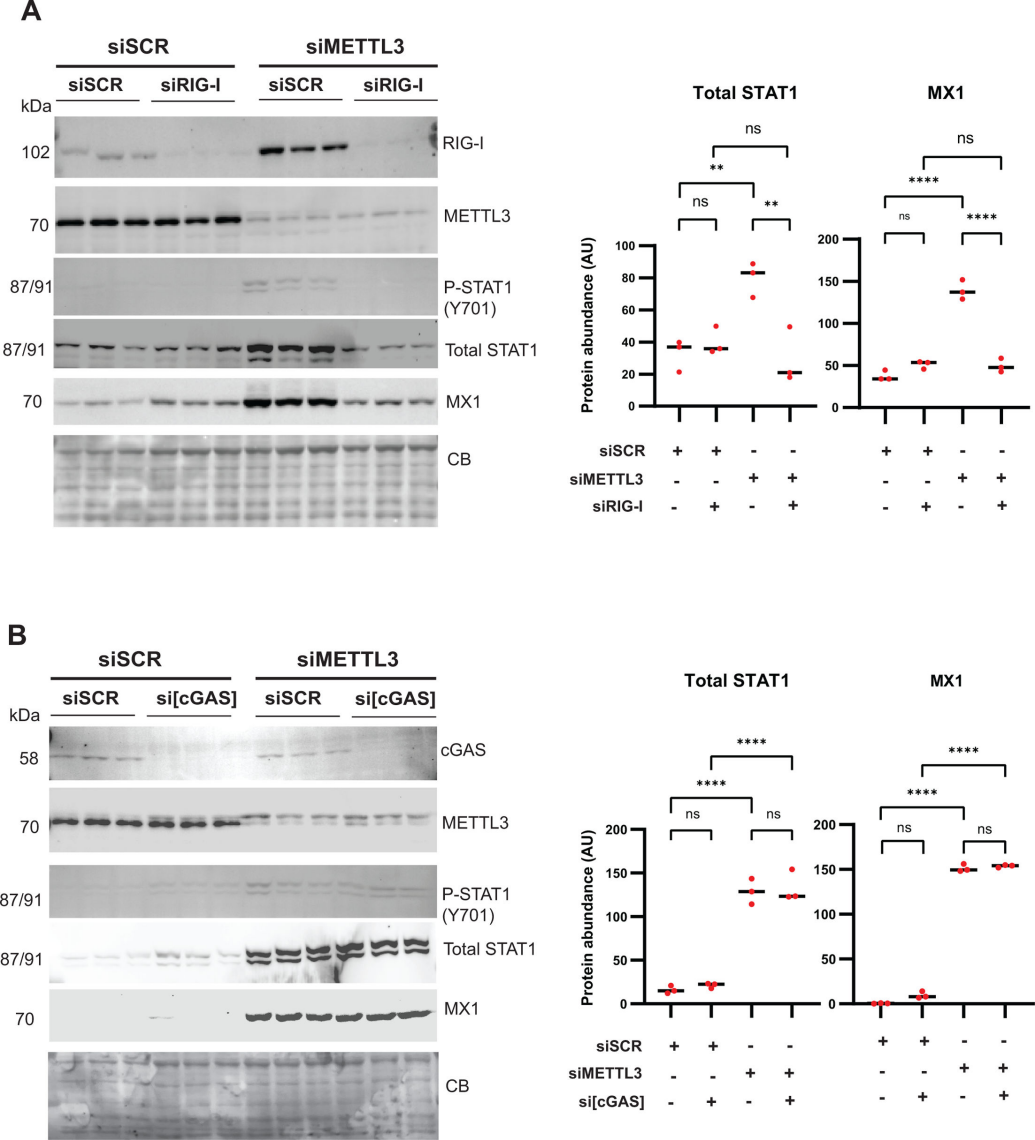
Effect of RIG-I or cGAS depletion on antiviral status and VSV replication in METTL3-depleted cells. SUIT-2 cells were transfected with either siSCR (**A and B**), siMETTL3 (**A and B**), siRIG-I (**A**), or si[cGAS] (**B**) for 48 h. Protein samples were analyzed by western blotting. Equal protein loading was verified by Coomassie Blue (CB) staining of the membranes. ImageJ software was used to quantify protein levels (**A and B**). Results were analyzed to determine significance using a one-way ANOVA with Fisher’s least significant difference test. ns, not significant, *P  <  0.05, **P  <  0.01, ***P  <  0.001, and ****P < 0.0001.

Several mechanisms can potentially explain how METTL3 depletion could activate ISG expression (including expression of STAT1 itself) via the JAK/STAT pathway, including RIG-I-mediated recognition of dsRNA (29, 31, 59–62) and cGAS-mediated recognition of RNA:DNA hybrid R-loops (63–65). To determine which cytosolic sensor mediates METTL3 depletion-dependent induction of the intrinsic antiviral state, we individually knocked down Retinoic Acid-Inducible Gene I (RIG-I) or cyclic GMP-AMP synthase (cGAS) in combination with siMETTL3 or siSCR (Fig. 6A and B). In siSCR SUIT-2 cells, knockdown of RIG-I or cGAS had no measurable effect on P-STAT1 (Y701), STAT1, and MX1 protein levels (Fig. 6A and B). Consistent with our above-mentioned findings, siMETTL3 SUIT-2 cells exhibited a significant increase in P-STAT1 (Y701), STAT1, and MX1 levels. However, the knockdown of RIG-I and METTL3 completely restored P-STAT1 (Y701), STAT1, and MX1 to the same basal levels as in siSCR SUIT-2 cells. In contrast, a combined knockdown of cGAS and METTL3 showed no difference in antiviral protein levels compared to METTL3 knockdown alone. Together, these data suggest that STAT1 phosphorylation (Y701), total STAT1, and ISG induction are dependent on the activation of RIG-I, but not cGAS, upon downregulation of METTL3. Notably, METTL3 depletion in SUIT-2 cells led to a dramatic increase in total RIG-I levels, which is not unexpected given that RIG-I is an ISG.

### Global transcriptome analysis of the effect of the METTL3 depletion on antiviral innate immune signaling in SUIT-2 and MIA PaCa-2 cells

To further understand the effect of METTL3 downregulation on the establishment of an intrinsic antiviral state in SUIT-2 cells, we conducted global transcriptome analysis comparing mRNA levels in uninfected SUIT-2 and MIA PaCa-2 treated with either siSCR or siMETTL3. SUIT-2 (functional antiviral signaling; responsive to siMETTL3) and MIA PaCa-2 (defective antiviral signaling; nonresponsive to siMETTL3) served as model cell lines representing these two different phenotypes of PDACs. While both SUIT-2 and MIA PaCa-2 are permissive to VSV-ΔM51 in the absence of METTL3 downregulation, siMETTL3 treatment had a dramatic inhibitory effect on VSV-ΔM51 replication in SUIT-2 cells but had no effect at all in MIA PaCa-2 (Fig. 1A and 4A). Our previous studies (9, 48, 49) and this study have shown that SUIT-2 cells have functional antiviral signaling (Fig. 2 to 5), while MIA PaCa-2 is defective in antiviral signaling (Fig. 4A), which might explain why siMETTL3 treatment was unable to establish an antiviral state in MIA PaCa-2.

To understand global gene expression fluctuations because of METTL3 depletion, total RNA was isolated from SUIT-2 and MIA PaCa-2 48 h after transfecting the cells with either siMETTL3 or siSCR and analyzed by poly-A enriched RNA sequencing (RNA-seq). This analysis revealed a global snapshot of gene expression before they were normally infected (Fig. 1 to 6) to help us understand the METTL3 depletion-mediated inhibition of VSV-ΔM51 replication. First, the Gene Ontology (GO) analysis was performed comparing significantly enriched biological processes between siMETTL3-treated SUIT-2 and siSCR-treated SUIT-2 (Fig. S1) or siMETTL3-treated MIA PaCa-2 and siSCR-treated MIA PaCa-2 (Fig. S2). For all GO panels, our significance cutoff was −log10(0.05). Importantly, among the top 40 biological processes globally enriched in siMETTL3-treated SUIT-2 (compared to siSCR-treated SUIT-2), 21 were involved in antiviral signaling (Fig. S1). However, in MIA PaCa-2, only two pathways related to antiviral signaling were globally enriched (Fig. S2). Volcano plots were used to identify individual genes significantly upregulated or downregulated within each of our comparisons: siMETTL3-treated SUIT-2 compared to siSCR-treated SUIT-2 (Fig. S3A) and siMETTL3-treated MIA PaCa-2 compared to siSCR-treated MIA PaCa-2 (Fig. S3B). The orange triangles represent all METTL genes and indicate the effect of siMETTL3 on the expression of METTL genes and confirm specific downregulation of METTL3 in both cell lines. Of note, METTL7A (alternative name: TMT1A; NM_014033.4), which is also a SAM-dependent methyltransferase that transfers methyl groups to thiol groups (66), appeared to be downregulated in the siMETTL3 condition in MIA PaCa-2, but not SUIT-2. However, all siRNAs targeting METTL3 do not complement any region in METTL7A mRNA. Therefore, we do not suspect there to be any off-target downregulation of METTL7A mRNA from our selected siRNA. On the other hand, we cannot rule out the possibility that METTL3 downregulation indirectly affects METTL7A expression. Since downregulated METTL7A was observed only in MIA PaCa-2, and not SUIT-2, we cannot rule out that the indirect downregulation of METTL7A may be associated with the inhibition of immune-related responses, hence why MIA PaCa-2 does not display a phenotype consistent with SUIT-2. The role of METTL7A would require a more careful examination in the future.

**Fig 7:**
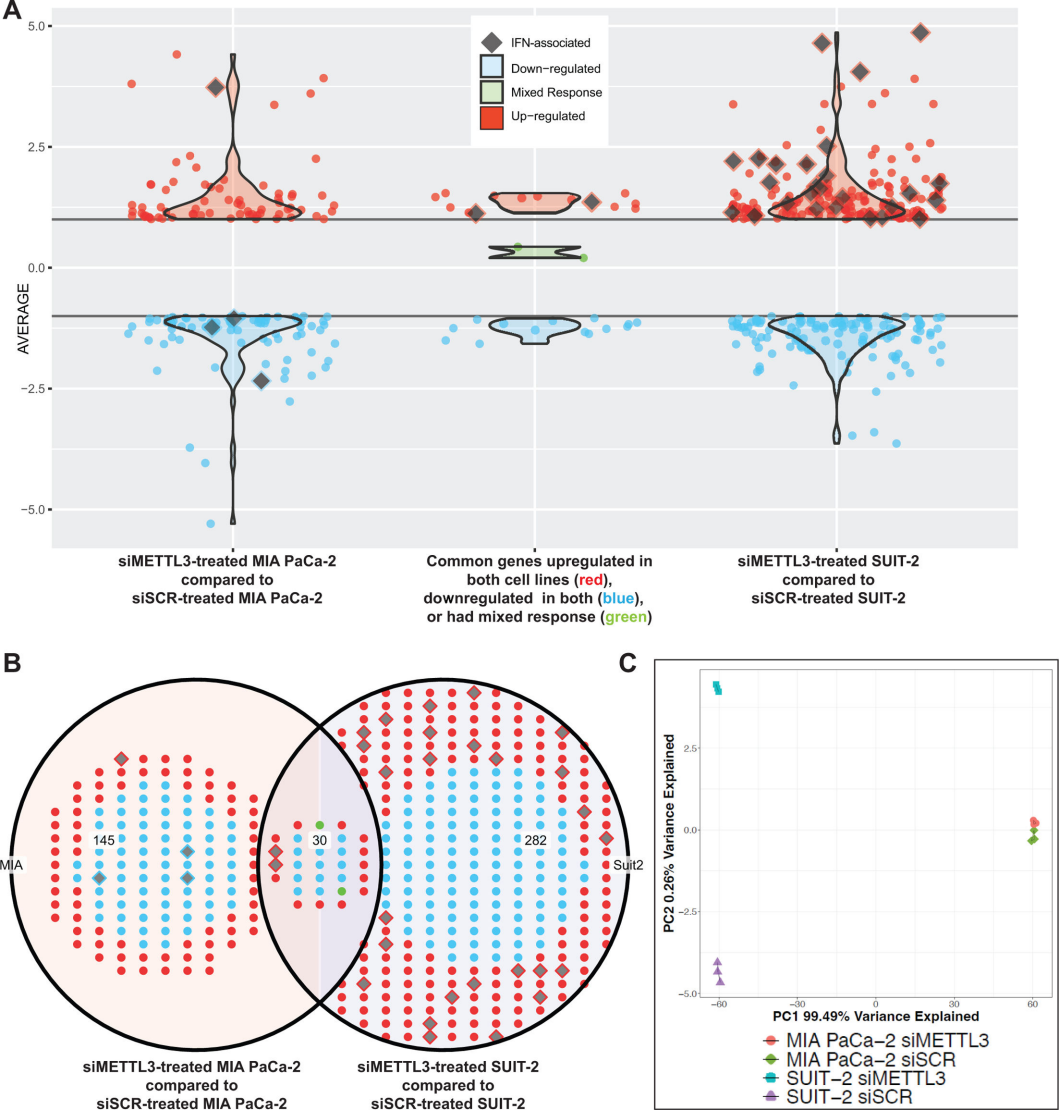
Comparison of differentially expressed total genes and genes of interest between groups from RNA-seq data. Red points represent upregulated genes, while blue indicate downregulated genes, and green points are an average of the two comparisons, and they show mixed response between the two comparisons and their respective siRNA controls. Gray diamonds indicate genes of interest found from a list by using the “Harmonizome” tool to focus on the “Interferon Signaling Gene Set Data set” of the Reactome Pathways data set. (**A**) Dot plot showing differentially expressed genes (DEGs) in MIA PaCa-2 (left), SUIT-2 (right), and shared DEGs between MIA PaCa-2 and SUIT-2 DEGs (center). Y-axis indicates the average log2foldchange. Violin plots represent the overall density of the differentially expressed genes within each group. (**B**) Venn diagram displaying DEGs in MIA PaCa-2 (left; 145), in SUIT-2 (right; 282), and shared DEGs between MIA PaCa-2 and SUIT-2 DEGs (center; 30). (**C**) Principal component analysis (PCA) plot showing variance between scramble siRNA and METTL3 siRNA in MIA PaCa-2 and SUIT-2. The rlog-transformed and DESeq2 normalized counts were used to generate the PCA, and the data were plotted with ggplot2. Bottom axis: PC1 99.49% variance. Left axis: PC2 0.26% variance. Four indicated conditions (*n* = 3 each) were compared.

To focus on antiviral genes, we used the “Harmonizome” tool and the “Interferon Signaling Gene Set Data set” of the Reactome Pathways data set to survey 194 candidate genes (67). These antiviral genes from this “Interferon Signaling Gene Set Data set” are represented by diamond shapes in the volcano plots for both comparisons (Fig. 7; Fig. S3). A total of 312 genes were differentially expressed in the SUIT-2 knockdown, while 175 differentially expressed genes (DEGs) were significant in the MIA PaCa-2 comparison. A total of 30 DEGs were shared between the two cell lines (Fig. 7). Interestingly, 26 IFN-associated genes were upregulated when METTL3 is knocked down in SUIT-2, while four genes were differentially expressed in MIA PaCa-2 (one upregulated and three downregulated). Two IFN genes Bone Marrow Stromal Antigen 2 (BST2) and Interferon Lambda Receptor 1 (IFNLR1) were upregulated in both cell lines with respect to siMETTL3. Unsurprisingly, most of the variance within our RNA-seq data comes from the difference between SUIT-2 and MIA PaCa-2 (PC1 99.49%) (Fig. 7C). However, METTL3 depletion within each cell line explains only 0.26% variance in the data (PC2). This shows that METTL3 knockdown has a relatively small change in the transcriptome, but that change is enough to have a major impact on VSV-ΔM51 replication, with the effect on the transcriptome being much bigger in SUIT-2 cells compared to MIA PaCa-2 cells.

**Fig 8:**
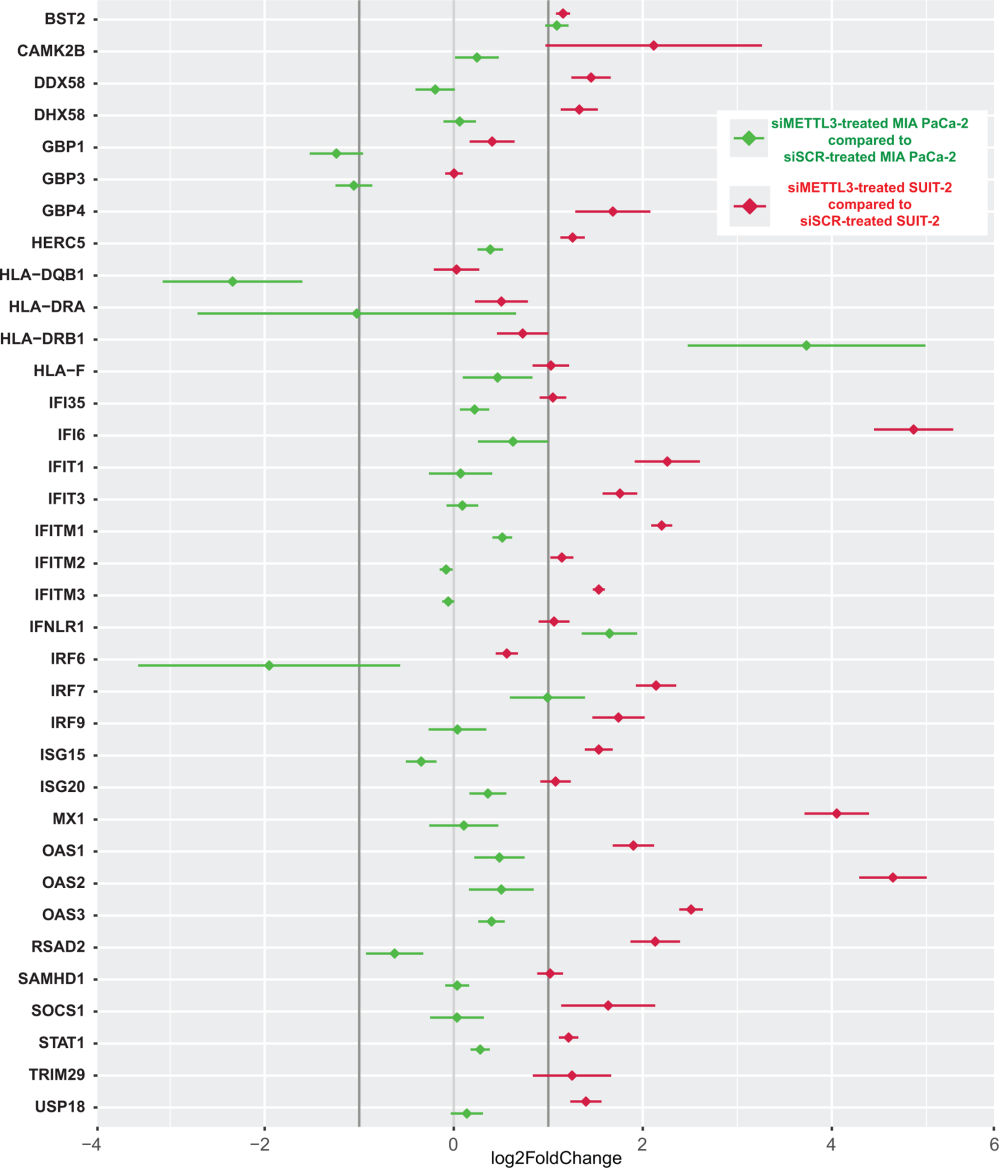
“Interferon Signaling Gene Set Data set” genes detected by RNA-seq analysis and found to be upregulated or downregulated or not changed in SUIT-2 or MIA PaCa-2. The “Interferon Signaling Gene Set Data set” of the Reactome Pathways data set was retrieved using the “Harmonizome” tool. Genes included in this column mean plot were detected by RNA-seq analysis and found to be upregulated or downregulated in either SUIT-2 or MIA PaCa-2 treated with siMETTL3 and compared to siSCR control. The *x*-axis indicates log2FoldChange and vertical gray bars are at −1 and 1 log2FoldChange. Red = SUIT-2 siMETTL3 (compared to SUIT-2 siSCR) and green = MIA PaCa-2 siMETTL3 (compared to MIA PaCa-2 siSCR). The data points and error bars shown represent the means and SEM of the means, respectively.

Notably, positive regulators of antiviral genes and effector ISGs are among the most upregulated antiviral-related genes in siMETTL3-treated SUIT-2 (compared to siSCR-treated SUIT-2) and include many well-known antiviral genes, such as DDX58 (DExD/H-box helicase 58; also known as RIG-I), DHX58 (DExH-Box Helicase 58; also known as LGP2), GBP4 (guanylate binding protein 4), IRF7 (interferon regulatory factor 7), IRF9, IFI6/35 (interferon alpha inducible protein 6/35), IFIH1 (Interferon Induced With Helicase C Domain 1, also known as MDA5), IFIT1 (interferon Induced Protein With Tetratricopeptide Repeats 1), IFIT2, IFIT3, IFITM1 (interferon induced transmembrane protein 1/2/3), IFITM2, IFITM3, IFNLR1, ISG15 (interferon stimulated gene 15), ISG20, MX1 (MX dynamin-like GTPase 1), OAS1 (2′,5′-oligoadenylate synthetase 1), OAS2, OAS3, RSAD2 (radical S-adenosyl methionine domain containing protein 2; also known as viperin), SAMHD1 (SAM And HD Domain Containing Deoxynucleoside Triphosphate Triphosphohydrolase 1), STAT1 (signal transducer and activator of transcription 1), and many others (Fig. 8; Fig. S3A). In contrast, siMETTL3-treated MIA PaCa-2 (compared to siSCR-treated MIA PaCa-2) showed very little upregulation in genes within this “Interferon Signaling Gene Set Data set” (Fig. 8; Fig. S3B), with the exception of BST2, HLA-DRB1 (major histocompatibility complex class II beta chain), IRF7, and IFNLR1. Interestingly, HLA-DRB1 was the only gene upregulated in siMETTL3-treated MIA PaCa-2 that was not also upregulated in siMETTL3-treated SUIT-2. Unlike siMETTL3-treated SUIT-2, five genes downregulated in siMETTL3-treated MIA PaCa-2 (compared to siSCR-treated MIA PaCa-2) were GBP1, GBP3, HLA-DQB1, HLA-DRA, and IRF6.

To examine the effect of METTL3 downregulation on specific antiviral pathways, we used the QIAGEN Ingenuity Pathway Analysis (https://digitalinsights.qiagen.com/IPA) (68). Figure S4A and S5A (for SUIT-2) and Fig. S4B and S5B (for MIA PaCa-2) display two pathways intimately associated with antiviral signaling: “Regulation of innate immune response by RNA sensing molecules” (Fig. S4) and “Interferon Signaling” (Fig. S5). RNA-helicase domain-containing proteins, DDX58 (also known as RIG-I), DHX58 (also known as LGP2), and IFIH1 (also known as MDA5), that are known to detect cytoplasmic double-stranded RNA (dsRNA) were upregulated in siMETTL3-treated SUIT-2 (compared to siSCR-treated SUIT-2) (Fig. S4A), but were only slightly elevated (DHX58/LGP2 and IFIH1/MDA5) or even slightly depleted (DDX58/RIG-I) in siMETTL3-treated MIA PaCa-2 (compared to siSCR-treated MIA PaCa-2) (Fig. S4B). Downstream transactivators, such as STAT1, IRF9, and IRF7, were highly upregulated, likely leading to the production of a subset of ISGs, such as IFI35, IFI6, IFITM1, IFITM2, IFITM3, IFIT1, IFIT3, IRF9, ISG15, MX1, and OAS1 in siMETTL3-treated SUIT-2 (compared to siSCR-treated SUIT-2) (Fig. S4A and S5A). In MIA PaCa-2, a similar trend was observed, but none of the genes within these pathways were strongly upregulated, other than IRF7 (Fig. S4B and S5B).

Previously, we characterized key differences in our PDAC cell lines' ability to respond to VSV-ΔM51, and a significant contribution to resistance was the ability of cells to produce type I IFN after viral infection (9). Surprisingly, IFNs (IFN-α, IFN-β, IFN-γ, or IFN-λ) and most of their receptors (except for IFNLR1, not shown in Fig. S5) were below detectable levels in SUIT-2 or MIA PaCa-2 (Fig. S4 and S5). Overall, our global transcriptome analysis shows that METTL3 depletion in SUIT-2 cells creates an intrinsic antiviral state characterized by an expression signature resembling IFN signaling.

### Role of secreted cytokines in METTL3 depletion-mediated activation of antiviral innate immune signaling

**Fig 9:**
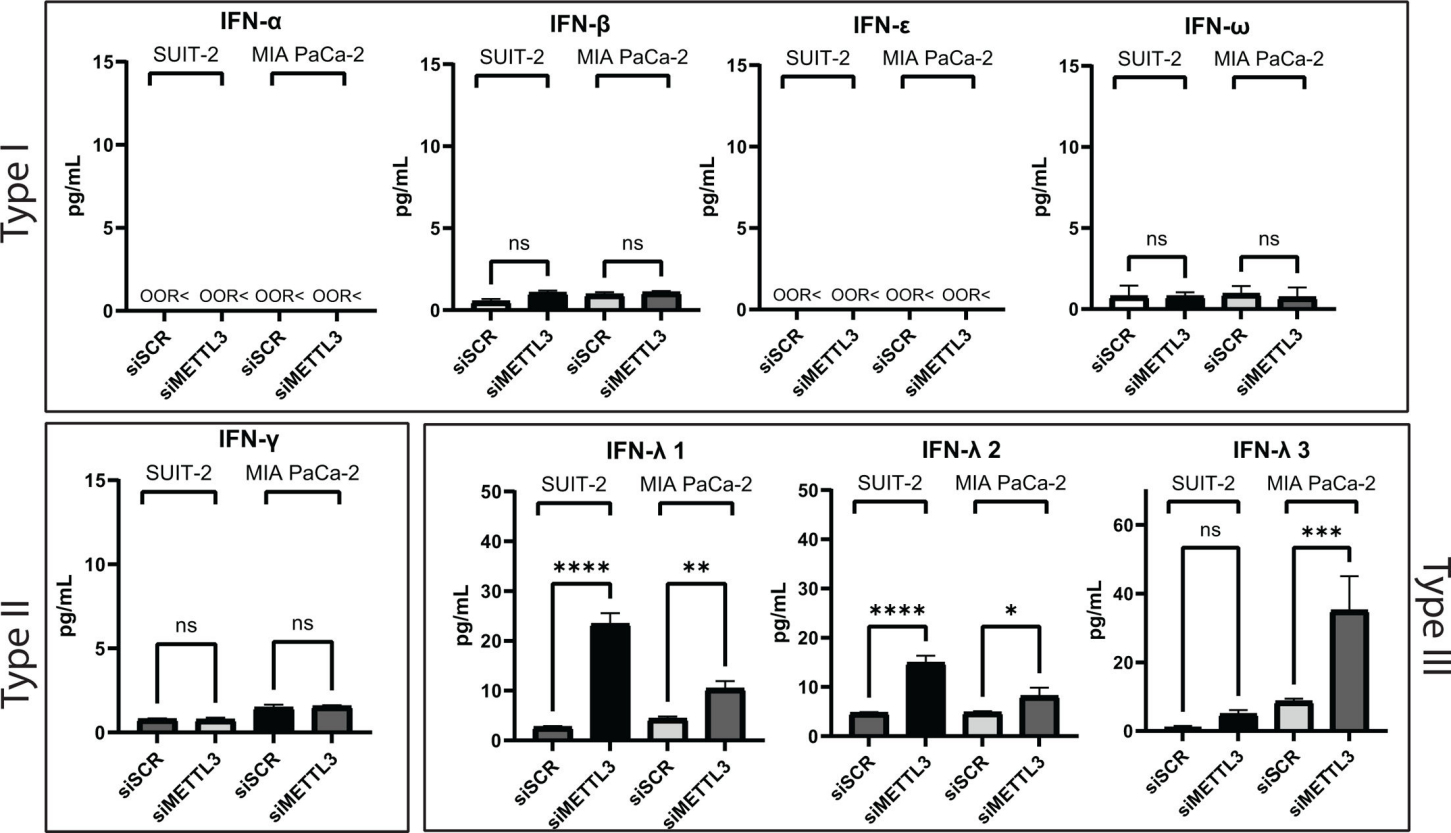
Human IFNs secreted in response to METTL3 depletion. Effect of METTL3 depletion on cytokine secretion by SUIT-2 and MIA PaCa-2. Cells were either transfected with siSCR or siMETTL3 for 48 h, and the cell culture medium was collected and sent to Eve Technologies for Human Interferon 9-Plex Discovery Assay Array (HDIFN9). Cytokines that were not found (below detection limit) shown as “OOR<”. The data points and error bars shown represent the means and SEM of the means, respectively. Results were analyzed to determine significance using the Student’s *t*-test comparing 1000 ng/mL for all cytokines. ns, not significant, *P  <  0.05, **P  <  0.01, ***P  <  0.001, and ****P < 0.0001.

Our RNA-seq analysis revealed many antiviral ISGs were upregulated in SUIT-2 (but not MIA PaCa-2) in response to METTL3 downregulation; however, none of the IFN mRNAs (type I IFN-α, type I IFN-β, type II IFN-γ or type III IFN-λ) were detected under any tested condition in SUIT-2 or MIA PaCa-2 (note that all RNA-seq samples were from uninfected cells). To examine the expression of IFNs at the protein level, the culture medium was collected 48 h post-transfection of uninfected SUIT-2 and MIA PaCa-2 cells with either siMETTL3 or siSCR and analyzed for the presence of IFNs using the Human Interferon 9-Plex Discovery Assay Array (HDIFN9, Eve Technologies) (Fig. 9). In agreement with the transcriptome analysis, type I IFN-α and IFN-ε were below detectable levels (marked as out-of-range “OOR<” in Fig. 9). Interestingly, although type I IFN-β and type II IFN-γ mRNAs were not detected by RNA-seq analysis, cytokine array analysis was able to detect IFN-β and IFN-γ at extremely low levels (around 0.1–1 pg/mL), and these levels were not altered in siMETTL3-treated cells (Fig. 9). Intriguingly, we observed statistically significant increases in siMETTL3-treated SUIT-2 (compared to siSCR-treated SUIT-2) for type III IFN-λ1 (also known as IL-29), and IFN-λ2 (also known as IL28A), and in siMETTL3-treated MIA PaCa-2 (compared to siSCR-treated MIA PaCa-2) for IFN-λ3 (IL28B) (Fig. 9).

**Fig 10:**
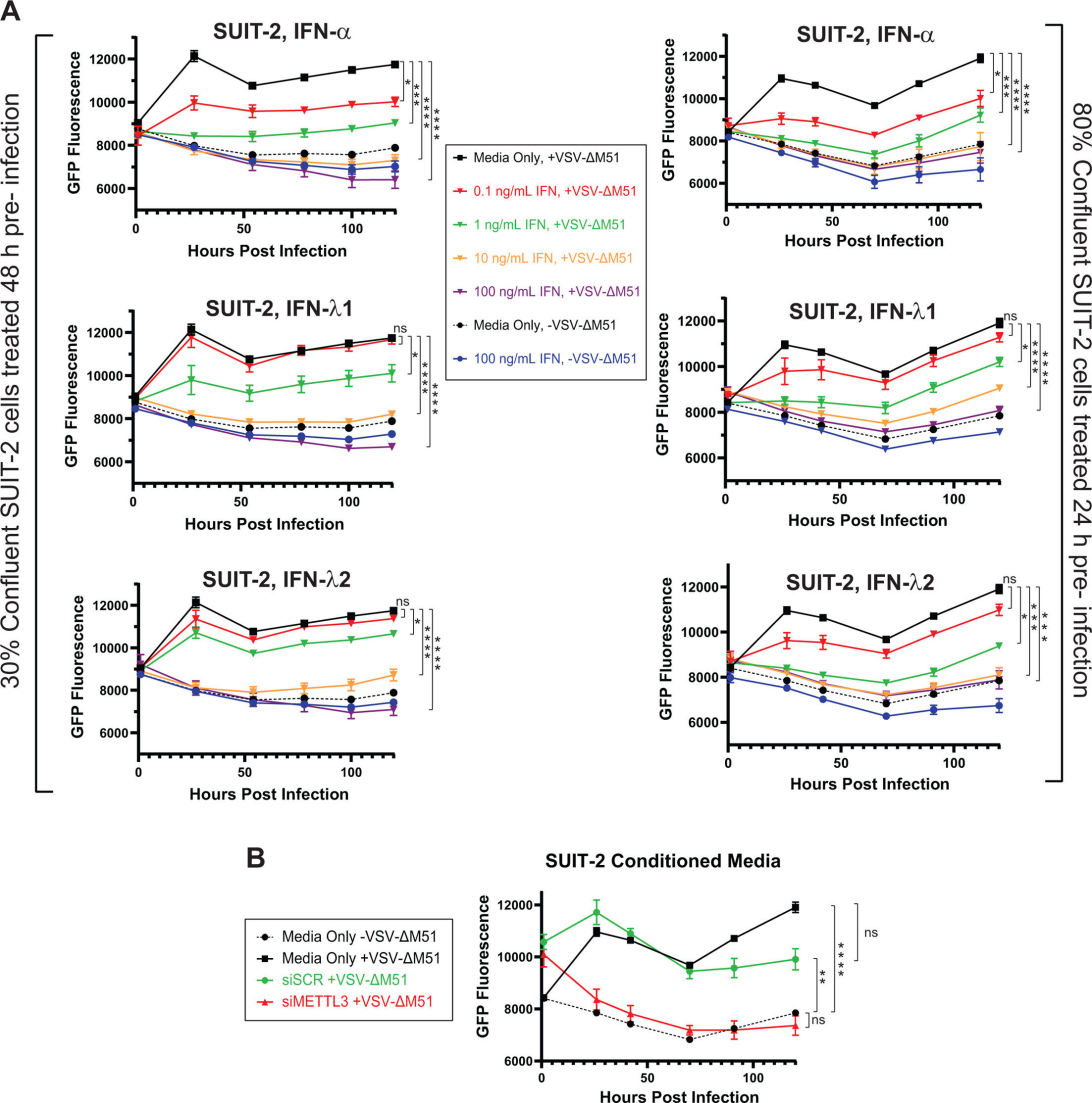
Responsiveness to IFNs in SUIT-2. (**A**) Responsiveness of SUIT-2 to human IFNs. Cells were seeded to target either 30% or 80% confluence the following day and treated with either human IFN-α, IFN-λ1, or IFN-λ2 either 24 h (80% confluent cells) or 48 h (30% confluent cells) before infection with VSV-ΔM51 at an MOI of 0.05 (based on VSV-ΔM51 titer on BHK-21). GFP fluorescence was measured between 1 and 120 h p.i. Results were analyzed to determine significance using the Student’s *t*-test comparing at 120 h p.i.: (1) Media only + VSV-ΔM51 vs 0.1 ng/mL IFN + VSV-ΔM51; (2) Media only + VSV-ΔM51 vs 1 ng/mL IFN + VSV-ΔM51; (3) Media only + VSV-ΔM51 vs 10 ng/mL IFN + VSV-ΔM51; (4) Media only + VSV-ΔM51 + VSV vs 100 ng/mL IFN + VSV-ΔM51. (**B**) SUIT-2 cells were seeded to reach ~35% confluency. Either scramble or METTL3 siRNA was transfected into cells. After 48 h post-transfection, the medium was collected. This medium was added to 80% confluent SUIT-2 cells 24 h before infection with VSV at MOI of 0.05 (based on VSV titer on BHK-21) or mock-treated. GFP fluorescence was measured between 1 and 120 h p.i. Results were analyzed to determine significance using the Student’s *t*-test comparing at 120 h p.i. (1) Media only + VSV-ΔM51 vs Media only − VSV-ΔM51; (2) Media only + VSV-ΔM51 vs siSCR + VSV-ΔM51; (3) Media only + VSV-ΔM51 vs siMETTL3 + VSV-ΔM51; (4) Media only − VSV-ΔM51 vs siMETTL3 + VSV-ΔM51. *P < 0.05, **P < 0.01, ***P < 0.001, ****P < 0.0001. ns, not significant.

We found the observed upregulation of IFN-λ1 and IFN-λ2 interesting, especially as both cell lines displayed the elevated expression of IFNLR1, one of the receptors for IFN-λ molecules. As we have never examined the responsiveness of PDAC cell lines to IFN-λ molecules, we decided to investigate a possible causative correlation between IFN-λ and the establishment of an antiviral state in siMETTL3-treated SUIT2 cells. In addition to different IFN-λ types, we also included in this experiment IFN-α, which served as a positive control previously shown to induce an antiviral state in most of the treated PDAC cell lines, including SUIT-2. SUIT-2 cells were treated with serial dilutions of IFN-λ1, IFN-λ2, or IFN-α, then infected at a MOI of 0.05, and viral growth kinetics were measured for 120 h p.i. (Fig. 10A). To simulate the siRNA transfection prior to infection conditions used throughout this study, we decided to test two alternative conditions: (i) 30%-confluent SUIT-2 cells were treated with IFNs for 48 h (mimics a scenario in which cells are exposed to IFN immediately after siRNA transfection) (left part of Fig. 10A), and (ii) 80%-confluent SUIT-2 cells were treated with IFNs for 24 h (mimics a scenario in which IFNs are produced 24 h post-transfection and cells are exposed to them for 24 h only) (right part of Fig. 10A). In agreement with our previous studies, SUIT-2 cells were highly responsive to IFN-α, which strongly inhibited VSV-ΔM51 replication at most tested IFN-α concentrations. Moreover, IFN-λ1 and IFN-λ2 also inhibited VSV-ΔM51 replication in SUIT-2 cells. There were no dramatic differences between responsiveness for the two tested scenarios (Fig. 10A).

**Fig 11:**
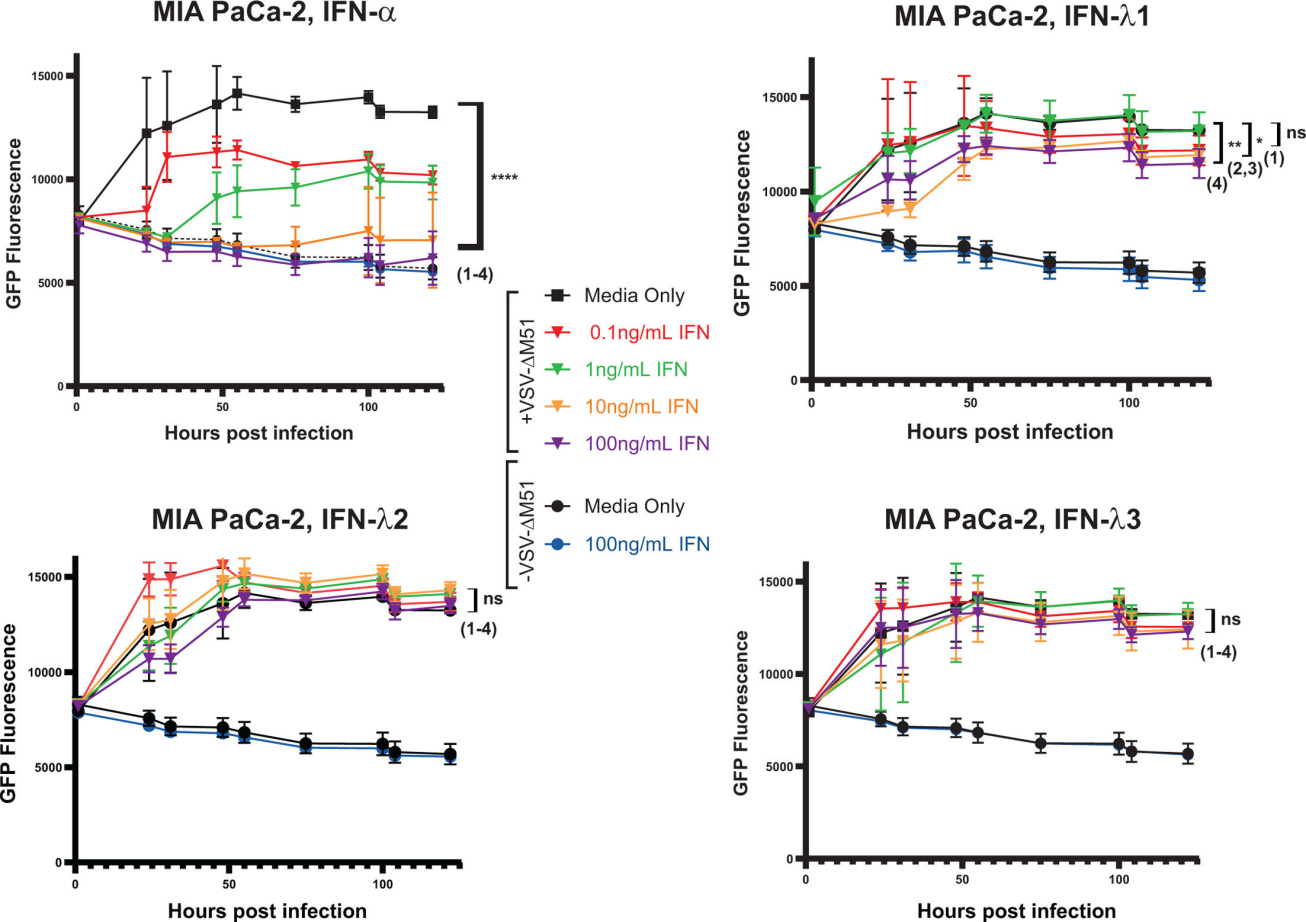
Responsiveness to IFNs in MIA PaCa-2. Responsiveness of MIA PaCa-2 to human IFNs. Cells were seeded to target 80% confluence the following day and treated with either human IFN-α, IFN-λ1, IFN-λ2, or IFN-λ3 for 24 h (80% confluent cells) before infection with VSV-ΔM51 at an MOI of 0.05 (based on VSV-ΔM51 titer on BHK-21). GFP fluorescence was measured between 1 and 120 h p.i. Results were analyzed to determine significance using the Student’s *t*-test comparing at 120 h p.i.: (1) Media only + VSV-ΔM51 vs 0.1 ng/mL IFN + VSV-ΔM51; (2) Media only + VSV-ΔM51 vs 1 ng/mL IFN + VSV-ΔM51; (3) Media only + VSV-ΔM51 vs 10 ng/mL IFN +VSV-ΔM51; (4) Media only + VSV-ΔM51 + VSV vs 100 ng/mL IFN + VSV-ΔM51. Results were analyzed to determine significance using the Student’s *t*-test compared at 120 h p.i. *P < 0.05, **P < 0.01, ***P < 0.001, ****P < 0.0001. ns, not significant.

Together, our data in Fig. 9 and 10A show that METTL3 depletion induces secretion of type III (but not type I or II) IFNs IFN-λ1 and IFN-λ2 in SUIT-2 cells (Fig. 9), and that SUIT-2 cells are responsive to antiviral effects of IFN-λ1 and IFN-λ2 (Fig. 10A). These data suggest that METTL3 depletion might activate the observed intrinsic antiviral state, at least in part, in an autocrine and paracrine fashion via production of antiviral cytokines, including (and likely not limited to) IFN-λ1 and IFN-λ2. To examine if METTL3 depletion inhibited VSV-ΔM51 replication due to secreted molecules of any kind, we first collected medium from SUIT-2 48 h after transfection with either siSCR or siMETTL3 and added this conditional medium to SUIT-2. 24 h later, we infected SUIT-2 with VSV-ΔM51 at a MOI of 0.05. Figure 10B shows that there was statistically significant inhibition of VSV-ΔM51 replication over the 120 h period post-infection. This suggests that METTL3 depletion leads to the production of secreted molecules that strongly inhibit VSV-ΔM51 replication (Fig. 10B). Although there was no observed VSV inhibition or increase in antiviral response in METTL3-depleted MIA PaCa-2 (Fig. 1, 2, 4, 7, and 8), we cannot ignore the significant increase in IFN-λ1–3 in MIA PaCa-2 after METTL3 downregulation, particularly IFN-λ3 (Fig. 9). To address the sensitivity of MIA PaCa-2 to IFN-λ1–3, MIA PaCa-2 cells were treated with serial dilutions of IFN-λ1, IFN-λ2, IFN-λ3, or IFN-α for 24 h, then infected at a MOI of 0.05, and viral growth kinetics was measured for 120 h p.i. (Fig. 11). IFN-α served as a positive control as IFN-α has previously been shown to induce an antiviral state in MIA PaCa-2. Consistent with previous studies, IFN-α treated MIA PaCa-2 resulted in significantly decreased VSV-driven GFP production at every concentration. In contrast, MIA PaCa-2 shows no sensitivity to IFN-λ2–3, as there is no significant decrease in virus replication even after treatment with 100 ng/mL of IFN. Whereas only starting at 10 ng/mL did VSV-driven GFP production begin to display significant decrease in MIA PaCa-2, although this concentration is 1,000 times more than the actual production of IFN-λ1 measured from the cytokine array (Fig. 9). Our data demonstrate that METTL3 depletion in SUIT-2 cells leads to the production of antiviral cytokines, particularly IFN-λ1 and IFN-λ2, which contribute to an intrinsic antiviral state. However, while MIA PaCa-2 cells also upregulate IFN-λ1–3 in response to METTL3 downregulation, they show limited sensitivity to these cytokines, likely explaining the lack of functional antiviral response in METTL3-depleted MIA PaCa-2.

### Effect of METTL3 depletion on replication of different VSV recombinants

**Fig 12:**
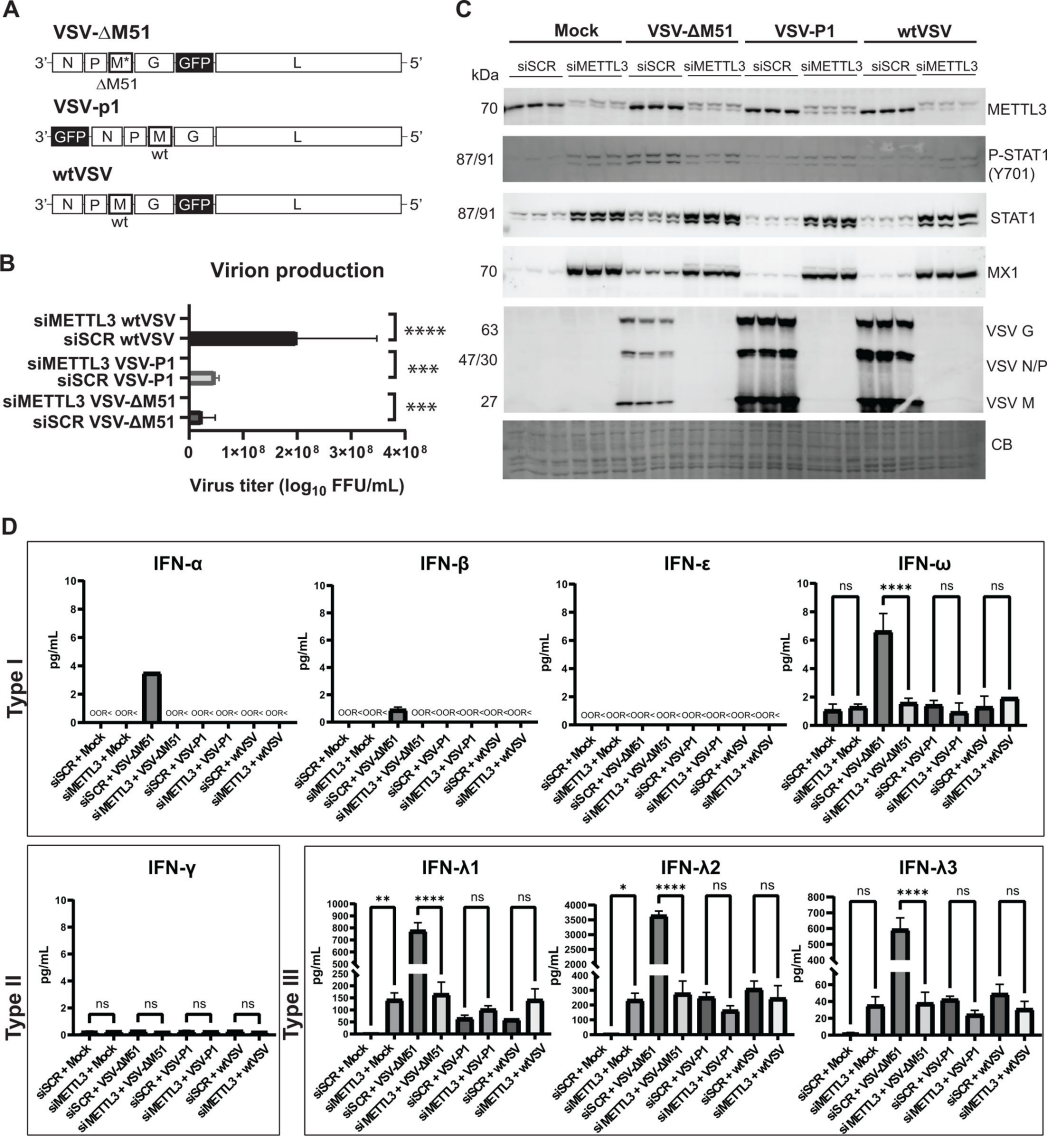
Effect of METTL3 depletion on replication of different VSV recombinants. (**A**) Schematic of VSV-ΔM51, VSV-Mwt-P1, and VSV-Mwt genomes used in this experiment. (**B**) *De novo* VSV virion production in siSCR and siMETTL3 treated SUIT-2 infected with VSV-ΔM51, VSV-Mwt-P1, or VSV-Mwt. (**C**) SUIT-2 cells were either transfected with siSCR or siMETTL3 for 48 h and then either mock infected or infected with either VSV-ΔM51, VSV-Mwt-P1, or VSV-Mwt at MOI 0.1 (based on VSV titer on BHK-21) for 24 h. Protein samples were analyzed by western blotting. Equal protein loading was verified by Coomassie Blue (CB) staining of the membranes. (**D**) VSV-ΔM51, VSV-Mwt-P1, or VSV-Mwt culture medium was collected 24 h p.i. and sent to Eve Technologies for Human Interferon 9-Plex Discovery Assay Array (HDIFN9). Cytokines that were not found (below detection limit) are indicated by “OOR<” (out of range). The data points and error bars shown represent the means and SEM of the means, respectively. Results were analyzed to determine significance using a one-way ANOVA with Fisher’s least significant difference test. ns, not significant, *P  <  0.05, **P  <  0.01, ***P  <  0.001, and ****P < 0.0001.

All the above-described experiments were conducted using a commonly used oncolytic VSV recombinant VSV-ΔM51. To examine whether METTL3 depletion also blocks replication of other VSV recombinants, we also tested two VSV recombinants encoding the wt M gene: VSV-Mwt and VSV-Mwt-P1 (Fig. 12A). Both viruses also encode the GFP reporter gene. VSV-Mwt has the same GFP insertion as VSV-ΔM51 but wt M gene. VSV-Mwt-P1 also has the wt M gene, but insertion of the GFP ORF at position 1 (P1) of the VSV genome results in slower viral replication kinetics. To further investigate the differences in viral replication between the three VSV recombinants (VSV-ΔM51, VSV-Mwt, and VSV-Mwt-P1), we harvested *de novo* virions produced 24 h after infection in siMETTL3 or siSCR-treated SUIT-2 cells. The collected viral particles were then used to infect BHK-21 cells at serial dilutions, and virus titers (FFU/mL) were quantified 14 h p.i. In siSCR SUIT-2 cells, virion production was much higher compared to dramatically lower yields in siMETTL3-treated SUIT-2 cells, with both attenuated VSV recominants (VSV-ΔM51 and VSV-Mwt-P1) producing fewer *de novo* virions than VSV-Mwt (Fig. 12B). As expected, both VSV recombinants with a wt M gene (VSV-Mwt and VSV-Mwt-P1) replicated more efficiently than VSV-ΔM51 in siSCR SUIT-2 cells (Fig. 12C). Also, in agreement with the role of the VSV-Mwt protein in inhibiting expression of antiviral genes, in siSCR SUIT-2 cells, VSV-ΔM51 induced higher levels of P-STAT1, STAT1, and MX1 compared to VSV-Mwt and VSV-Mwt-P1. However, in siMETTL3 SUIT-2 cells, replication was dramatically inhibited regardless of the VSV recombinant used. P-STAT1, STAT1, and MX1 levels were similar across all siMETTL3 groups (Mock, VSV-ΔM51, VSV-Mwt, and VSV-Mwt-P1), with no further increase in P-STAT1, STAT1, or MX1 was observed after infection with any virus, compared to mock. The absence of changes in protein levels and the lack of successful viral replication (indicated by western blot and GFP microscopy) in the siMETTL3 groups, regardless of the VSV-M variant, further support the conclusion that downregulating METTL3 in SUIT-2 cells generates sufficient antiviral factors to combat infection with all tested recombinant VSVs (Fig. 12C).

Figure 9 showed specific induction of type III (but not type I or type II) IFNs in uninfected siMETTL3-treated SUIT-2 and MIA PaCa-2 cells. After noticing such a dramatic effect of siMETTL3 on all recombinant VSVs, we wanted to examine if there was further induction of IFNs in virus-infected siMETTL3 SUIT-2 cells compared to siSCR SUIT-2 cells. To examine the levels of secreted IFNs, SUIT-2 cells were transfected with siSCR or siMETTL3 for 48 h, then infected with VSV-ΔM51, VSV-Mwt, or VSV-Mwt-P1 at MOI 0.1 for 24 h, the medium was collected and analyzed for the presence of IFNs using the Human Interferon 9-Plex Discovery Assay Array (HDIFN9, Eve Technologies) (Fig. 12D). Noticeably, VSV-ΔM51 infected siSCR SUIT-2 cells showed the highest induction of IFN-α, IFN-β, IFN-ω, and IFN-λ1–3 compared to VSV-Mwt and VSV-Mwt-P1, which can be attributed to the inability of the mutant VSV-ΔM51 protein to block antiviral expression. However, in siMETTL3, there was no further induction of IFNs past the level seen in the mock-infection condition for any of the three tested VSV recombinants. We conclude that siMETTL3 SUIT-2 cells do not require additional IFN production to combat VSV infection, resulting in little to no change in cytokine production post-infection.

### METTL3 depletion in different PDAC cell lines and non-PDAC cells

**Fig 13:**
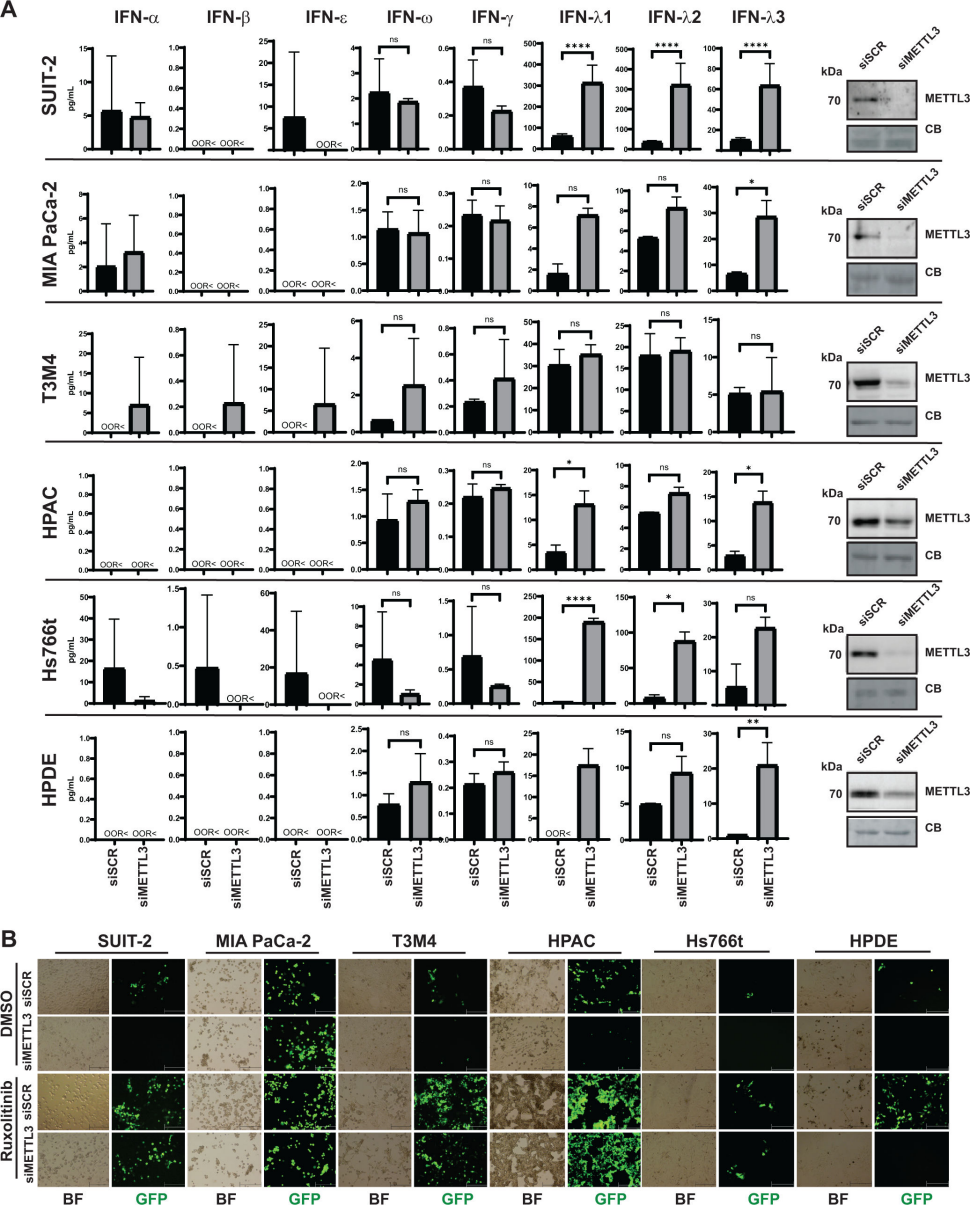
Effect of METTL3 depletion on five PDAC cell lines and one pancreatic cell line. Cells were either transfected with siSCR or siMETTL3 for 48 h. (**A**) The cell culture medium was collected and sent to Eve Technologies for Human Interferon 9-Plex Discovery Assay Array (HDIFN9). Cytokines that were not found (below detection limit) shown as “OOR<”. The data points and error bars represent the means and SEM of the means, respectively. Results were analyzed to determine significance using the Student’s *t*-test comparing 1000 ng/mL for all cytokines. ns, not significant, *P  <  0.05, **P  <  0.01, ***P  <  0.001, and ****P < 0.0001. Each cell line has the respective western blot analysis of METTL3 with a representative from siSCR and siMETTL3 condition. Equal protein loading was verified by Coomassie Blue (CB) staining of the membranes. (**B**) Cells were pre-treated with Ruxolitinib (or DMSO control) for 24 h after transfection with either siSCR or siMETTL3. 24 h after Ruxolitinib treatment, cells were infected with VSV-ΔM51 (MOI 0.5; based on VSV titer on BHK-21) and Brightfield (BF) and GFP microscopy images were taken with Nikon camera at 24 h p.i. using 100× objective.

In Fig. 1, we demonstrated a significant reduction in viral protein production across most PDAC cell lines following METTL3 downregulation, with SUIT-2 cells showing that this inhibition is driven by enhanced antiviral signaling. However, this observation has thus far been limited to SUIT-2 cells. To explore whether a similar mechanism occurs in other cell lines, we analyzed, in addition to SUIT-2 and MIA PaCa-2, three additional PDAC cell lines (Hs766t, HPAC, and T3M4), an immortalized HPDE cell line, and a human lung adenocarcinoma cell line (A549). Culture medium was collected 48 h after transfection with either siSCR or siMETTL3 and analyzed for the presence of IFNs using the Human Interferon 9-Plex Discovery Assay Array (HDIFN9, Eve Technologies) (Fig. 13A). Consistent with the findings in Fig. 9 and 12, we observed an increase in IFN-λ1 and IFN-λ2 in SUIT-2 cells and IFN-λ3 in MIA PaCa-2 cells. Similarly, both HPAC and Hs766t cells exhibited significant increases in IFN-λ1, along with either IFN-λ3 or IFN-λ2, respectively (Fig. 13A). Notably, siMETTL3 also led to elevated levels of IFN-λ1, IFN-λ2, and IFN-λ3 in an immortal HPDE cell line. In contrast, T3M4 cells showed a trend towards increased levels of IFN-α, IFN-β, IFN-ε, IFN-ω, and IFN-γ, although IFN-ω and IFN-γ increases were not statistically significant, and no changes were observed in IFN-λ levels. To address if the IFN differences observed were dependent on JAK/STAT signaling, all cells were pre-treated with ruxolitinib for 24 h after transfection with either siSCR or siMETTL3 and then, 24 h later, infected with VSV-ΔM51 at an MOI of 0.5 (based on BHK-21 titer) (Fig. 13B). As noted previously, ruxolitinib is a reversible drug; therefore, it was re-added back after VSV-ΔM51 infection. As also seen in Fig. 5E, VSV-driven GFP expression was restored in siMETTL3 SUIT-2 treated with ruxolitinib (Fig. 13B). MIA PaCa-2 showed similar levels of VSV-driven GFP in each condition, regardless of ruxolitinib or siRNA treatments, which is unsurprising as MIA PaCa-2 is highly permissive to VSV-ΔM51 and has not displayed a competent antiviral response, even after siMETTL3 transfection. In T3M4, HPAC, Hs766t, and HPDE, VSV-driven GFP expression decreased following siMETTL3 transfection. Furthermore, in the siSCR condition, VSV-driven GFP expression increased upon ruxolitinib treatment in all these cell lines. However, only in T3M4, HPAC, and Hs766t cells did ruxolitinib treatment restore VSV-driven GFP expression in the siMETTL3 condition, while no restoration of GFP levels was observed in immortal HPDE cells transfected with siMETTL3. In general, our findings show that the downregulation of METTL3 suppresses virus replication by enhancing antiviral signaling in multiple pancreatic cancer cell lines and a nonmalignant pancreatic cell line, with JAK/STAT signaling being a critical factor in this process in PDAC cell lines.

**Fig 14:**
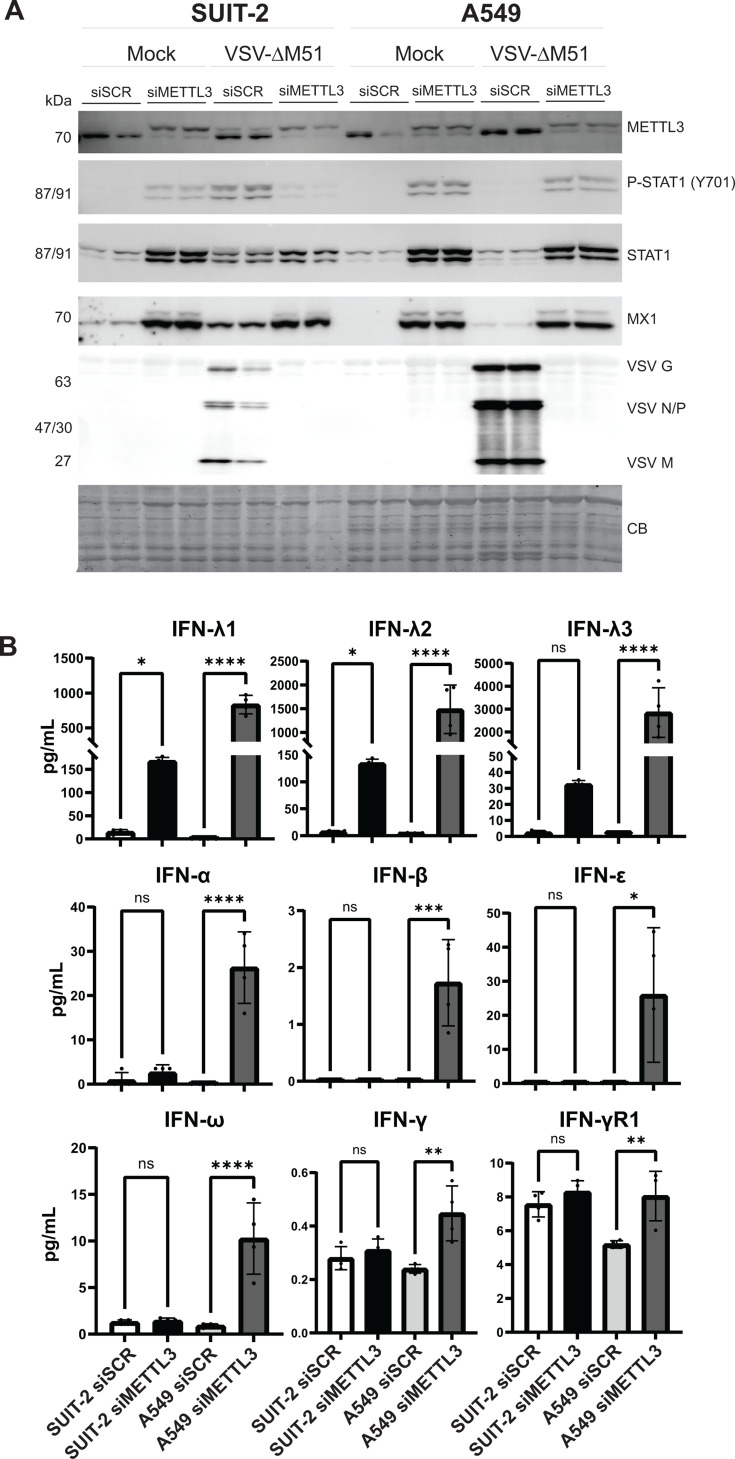
Effect of METTL3 depletion on nonpancreatic cell line. Cells were either transfected with siSCR or siMETTL3 for 48 h, (**A**) then infected with either mock or VSV (MOI 0.1; based on VSV titer on BHK-21) for 24 h. Protein samples were analyzed by western blotting. Equal protein loading was verified by Coomassie Blue (CB) staining of the membranes. (**B**) The cell culture medium (before infection and 48 h after transfection) was collected and sent to Eve Technologies for Human Interferon 9-Plex Discovery Assay Array (HDIFN9). Cytokines that were not found (below detection limit) shown as “OOR<”. The data points and error bars shown represent the means and SEM of the means, respectively. Results were analyzed to determine significance using the Student’s *t*-test comparing 1000 ng/mL for all cytokines. ns, not significant, *P  <  0.05, **P  <  0.01, ***P  <  0.001, and ****P < 0.0001.

Given that our data suggest METTL3 downregulation inhibits virus replication through antiviral cytokines in PDAC, and this effect is also observed in nonmalignant pancreatic cells, we sought to determine if a similar mechanism can be observed in a non-PDAC cell line. To investigate this, we tested A549, a human lung carcinoma cell line, widely used to study antiviral innate immunity, as it retains an intact innate immune system despite immortalization. We transfected both SUIT-2 and A549 cells with either siSCR or siMETTL3, followed by infection with VSV-ΔM51 at an MOI of 0.1 (based on BHK-21 titer). In both SUIT-2 and A549, VSV-ΔM51 replication was successful in siSCR conditions in both cell lines, more so in A549, and both cell lines showed increased P-STAT1, total STAT1, and MX1 in mock conditions transfected with siMETTL3 (Fig. 14A). Interestingly, P-STAT1 was heightened only in siMETTL3 conditions in A549 regardless of VSV infection. In SUIT-2, P-STAT1 contrasts with that shown in A549, particularly in the infected conditions. To investigate the response in A549 compared to SUIT-2 upon METTL3 downregulation (before infection), the medium was collected 48 h after transfection with either siSCR or siMETTL3 and analyzed for the presence of IFNs using the Human Interferon 9-Plex Discovery Assay Array (HDIFN9, Eve Technologies) (Fig. 14B). A549 shows a significantly heightened induction of cytokines in every IFN class, especially IFN-λ1–3. Consistent with Fig. 9 and 13A, SUIT-2 demonstrates the main induction of cytokine production as IFN-λ1–2 with no significant difference in type I or II IFNs. In conclusion, our data suggest that the downregulation of METTL3 inhibits virus replication through the activation of antiviral cytokines in not only PDAC and an immortalized HPDE cell line but also in the A549 lung carcinoma cell line, where METTL3 downregulation leads to heightened cytokine production, particularly IFN-λ1–3. While both SUIT-2 and A549 cells show increased antiviral signaling in response to METTL3 knockdown, the specific cytokine profiles differed, with A549 exhibiting a broader induction across all IFN classes.

## DISCUSSION

PDAC is one of the deadliest types of cancer, largely because the disease is often diagnosed at a late stage, which severely limits effective treatment options and leads to a rapid development of resistance to standard therapies (1, 2). OV therapy is a promising approach against PDAC via the use of replication-competent “oncolytic viruses” designed to specifically target and destroy cancer cells while sparing healthy ones. To create effective OV therapies for PDAC, it is crucial to identify negative and positive host factors controlling OV replication in PDAC cells (10). While negative host factors (such as various host proteins associated with type I IFN signaling) typically play an important role in restricting viral replication, positive host factors are required for any stage of viral replication or play a role in inhibiting host responses to viral infection (69). Here, we demonstrate that the cellular protein METTL3, which was previously shown to promote pancreatic cancer cell proliferation, invasion, and resistance to chemotherapy, plays a positive role in OV replication in most of the tested human PDAC cell lines.

While METTL3 was found to be upregulated (70) and oncogenic (23–25) in PDAC in previous studies, its role in virus replication in PDAC cells was unclear. In this study, we investigated the effects of siRNA-mediated depletion of METTL3 on VSV-ΔM51 replication in a set of 10 different human PDAC cell lines. In addition, we examined the impact of the pharmacological inhibition of METTL3 in two PDAC cell lines, SUIT-2 and MIA PaCa-2. Our data showed that targeting METTL3 increased resistance to VSV-ΔM51 in PDAC cell lines with functional innate antiviral signaling (most of the tested PDACs) but had no effect in PDAC cell lines with defective innate antiviral signaling (MIA PaCa-2 and Capan-1). The observed phenotypes were not determined by the basal level of METTL3 expression. It is also important to note that there were no PDAC cell lines where METTL3 depletion enhanced VSV-ΔM51 replication.

METTL3 depletion not affecting VSV-ΔM51 replication in PDAC cell lines with defective antiviral signaling is intriguing, as METTL3 globally impacts many cellular processes, including RNA stability, splicing, translation, and decay (11–14), and m6A is present on over 7,600 genes and commonly known as one of the most prevalent modifications on eukaryotic RNA (71). In contrast, METTL3 depletion dramatically inhibited VSV-ΔM51 replication in PDAC cell lines with functional innate antiviral signaling, defining METTL3 as a positive host factor that inhibits antiviral responses. Our data indicate that METTL3 depletion leads to the onset of an intrinsic antiviral state. Immune responses are categorized into adaptive and innate immunity. Innate immunity includes intrinsic immunity, which consists of constitutively active antiviral mechanisms in uninfected cells, while induced immunity is triggered by infection. Intrinsic immunity is defined by the continuous expression of ISGs, which establish a restrictive environment for viral infection (72). Moreover, some studies consider intrinsic immunity as a third component of the immune system. In general, the establishment of an antiviral state is characterized by the activation and upregulation of hundreds of ISGs and can be divided into three functional groups regarding their effect on viral replication: antiviral effectors, positive regulators, and negative regulators (73, 74). Antiviral effectors inhibit viral infection by directly targeting the replicating virus, while positive regulators (sensors and transactivators) increase the immune response by enhancing recognition or innate immune signaling, and negative regulators decrease or terminate innate immune signaling to keep the immune response in tight control (73, 74). In our data, we identified numerous positive regulators and antiviral effectors upregulated in response to METTL3 depletion. Thus, DDX58 (also known as RIG-I), IFIH1 (also known as MDA5), and DHX58 (also known as LGP2), IRF7, IRF9, and STAT1 are critical positive regulators for the expression of effector genes known to directly inhibit viral replication (GBP4, IFITM3, IFITM1, IFITM2, IFITM3, IFI35, IFIT1, IFIT3, IFI6, ISG15, MX1, OAS1, RSAD2, SAMHD1, etc.) (56, 73, 74). Interestingly, a similar subset of constitutively expressed ISGs was previously identified in our laboratory in untreated “superresistant” PDAC cell lines, HPAF-II and Hs766T (48). HPAF-II and Hs766T are the most resistant PDAC among those tested, due to their intrinsic immunity. They express high levels of many ISGs, which remain unchanged even after virus infection (48). In this study, upon METTL3 downregulation in SUIT-2, we observed a similar ISG expression pattern. Of note, the known antiviral transactivator STAT1 and a major antiviral effector MX1 were expressed at such high levels after METTL3 knockdown that VSV infection did not induce any further increase in the expression of STAT1 or MX1.

VSV and other NNS RNA viruses have been shown to acquire m6A in viral RNA as a common strategy to evade host innate immune responses (31). Studies show that depleting METTL3 negatively affects VSV and other NNS RNA virus replication (29, 31, 75). One study showed that METTL3-deficient mice infected with VSV had an increase in activated IRF3, TBK1, and STAT1 (29). In HeLa cells, VSV infection caused METTL3 to translocate to the cytoplasm, enriching viral RNA with m6A and preventing recognition by immune sensors like RIG-I and MDA5 (29, 31). A related study found that VSV infection increased the interaction between METTL3 and the RNA helicase protein DDX5 (75). That study proposed that DDX5 regulates the interaction between METTL3 with its adaptor, METTL14, after infection (75). DDX5 was shown to negatively regulate the antiviral response by promoting m6A modifications on antiviral mRNAs, leading to their decay via YTHDF2 (75). To date, METTL3 and m6A have been studied primarily in the context of viral m6A enrichment and cellular responses to infection under conditions that alter m6A levels on viral transcripts.

Although our study does not rule out the contributions of these mechanisms to METTL3 depletion-mediated inhibition of VSV replication, we offer a new perspective by demonstrating the impact of METTL3 depletion on intrinsic antiviral immunity and its role in blocking VSV replication. In agreement with previous studies, our data confirm that METTL3 is a positive host factor of VSV replication and acts as a dampener of antiviral responses in PDAC cells with functional antiviral signaling. However, our study proposes an alternative explanation. We propose that METTL3 depletion activates antiviral signaling primarily by modifying the expression of cellular mRNAs encoding antiviral regulator and effector genes, which happens independently of viral infection (and, as we show, even before virus infection). We show that the establishment of this virus-independent, intrinsic antiviral state in METTL3-depleted PDAC cells is dependent on RIG-I (but not cGAS) and is marked by type-III (but not type I or II) IFN secretion and constitutive overexpression of antiviral sensors [RIG-I (DDX58), MDA5 (IFIH1), and LGP2 (DHX58)], transactivators (STAT1, IRF7, and IRF9), and a diverse subset of antiviral effectors, including MX1, OAS1/2/3, and IFIT1/3. In agreement with the role of secreted type III IFNs in this mechanism, ruxolitinib (highly specific JAK1/JAK2 inhibitor) was able to dramatically suppress the effect of METTL3 depletion-mediated ISG upregulation and subsequent inhibition of VSV replication, implicating the involvement of JAK1 and/or JAK2 in this phenotype. However, as VSV protein accumulation was still decreased in ruxolitinib-treated siMETTL3-transfected cells compared to ruxolitinib-treated siSCR-transfected cells, we cannot rule out that some alternative JAK1/2-independent pathways could also potentially contribute to this phenotype. Additionally, as seen in Fig. 13B, ruxolitinib did not restore any VSV-driven GFP production in a METTL3-depleted immortal HPDE cell line. We found this quite interesting as this observation may bring up additional points to consider. Nontransformed pancreatic tissue and cell lines are expected to exhibit strong activation of antiviral signaling. When the expression of antiviral genes is further elevated, such as with the downregulation of METTL3, the inhibition of JAK1/2 not enhancing VSV replication may potentially be due to sufficient abundance of antiviral genes already within the cell before treatment with ruxolitinib. On the other hand, sustaining antiviral activity is not conducive to maintaining cancer cells; therefore, HPDE may obtain alternative antiviral signaling pathways that are simply inactive in many PDAC cell lines (76).

Earlier research highlights several potential mechanisms through which the depletion of METTL3 may trigger antiviral signaling activation, including the increased abilities of RIG-I to recognize its RNA ligands (RNA containing m6A modifications bound RIG-I poorly) (59), increased formation of endogenous dsRNAs recognizable by RIG-I (77), response to activation of endogenous retroviruses (ERVs) (78), stabilization of IFN-β mRNA (79, 80), and cGAS-mediated response to increased accumulation of RNA:DNA hybrids (R-loops) in METTL3 depleted cells (63, 65). R-loops are RNA:DNA hybrid and unpaired single-stranded DNA that are a source of genome instability. m6A-enriched R-loops recruit YTHDF2 to promote RNA degradation and conversely result in the accumulation of R-loops upon METTL3 depletion (63). Ku70 protein, which is associated with IRF1 and IRF7 (81), recognizes cytosolic DNA and RNA:DNA hybrids from unrepaired R-loops exported into the cytoplasm. This may lead to the activation of the cGAS/STING pathway and may be responsible for the IFN-independent upregulation of an IRDS-associated subset of ISGs. Our findings indicate that the loss of cGAS does not reduce the levels of antiviral proteins, suggesting that cGAS may not be essential for the METTL3-mediated antiviral response in SUIT-2 cells. While this observation holds, we cannot completely rule out the possibility that METTL3 depletion might contribute to the formation of R-loops in these cells, which could influence the antiviral response in a cGAS-independent fashion. Future studies will be needed to explore these alternative pathways and identify the full range of factors involved in the antiviral response in METTL3-depleted cells.

Our data indicate that the observed establishment of virus-independent intrinsic antiviral state was RIG-I dependent. RIG-I (DDX58) is a cytoplasmic pattern recognition receptor (PRR) that plays a crucial role in the innate immune system. It detects viral RNA in the cytoplasm, particularly RNA with 5′-triphosphates or double-stranded regions. RIG-I activates downstream signaling pathways, including those involving MAVS (mitochondrial antiviral signaling protein) and the NF-κB and IRF3 transcription factors, to orchestrate the host’s defense against viral infections. Different ways of RIG-I activation in METTL3-depleted cells have been documented in earlier studies. Thus, *in vitro* synthesized RNA with m6A modifications was shown to be bound poorly to RIG-I, failing to trigger the conformational change necessary for RIG-I activation or to induce an innate immune response (59). A human metapneumovirus (HMPV) infection model showed that HMPV without m6A modifications was more effectively captured by RIG-I (but not by MDA5), resulting in RIG-I activation and oligomerization (30). The study concluded that m6A modifications inhibit type I IFN production by shielding viral RNA from being detected by RIG-I, both *in vitro* and *in vivo* (30). Several other studies have found that m6A modifications similarly influence the recognition of various viruses, including HBV, HCV, HIV-1, MeV, SeV, VSV, and SARS-CoV-2 (29, 31, 60–62). Some of these studies revealed that m6A-modified viral RNAs attract reader proteins YTHDF2 (YT521-B homology domain-containing family protein 2) and YTHDF3, which prevent RIG-I from sensing the viral RNA (31, 60). The increased protein-bound RNA facilitated by m6A resulted in a decrease in cellular double-stranded RNA structures that are crucial for RIG-I recognition (29, 82). Aside from YTH-domain containing reader proteins, hnRNPC (heterogeneous nuclear ribonucleoprotein C) belongs to another class of m6A reader proteins and can in turn alter the secondary structure, known as “structural switches” (83). m6A modifications can induce structural changes in RNA by altering how hnRNPs bind to it. When hnRNPs recognize m6A sites, they can cause the RNA to fold differently, which may expose or hide other functional regions, impacting mRNA overall conformation and stability (83).

Interestingly, T3M4 displayed a unique IFN production profile compared to SUIT-2, HPAC, and Hs766t, which are most comparable as all are PDAC cell lines that show an increased antiviral response after siMETTL3 transfection (Fig. 13A). Even HPDE, which is most representative of normal pancreatic ductal cells, induced significantly more type III IFNs after METTL3 downregulation. The unique combination and fine-tuning of cytokine induction and overall antiviral gene expression between cell lines are no surprise, as PDAC cell lines are notoriously heterogeneous in ploidy (50), epigenetic profiles (84, 85), proteostasis (86), and overall gene regulation (87). Additionally, knockdown success varied between each cell line, which may potentially play a role in the abundance of cytokine production; however, it is unlikely to have contributed to which cytokine was produced. Type III IFNs were also significantly upregulated in METTL3 downregulated MIA PaCa-2, although VSV replication was not changed regardless of this. It is likely that METTL3 downregulated cell lines not displaying the observed heightened antiviral phenotype is due to the inability to respond to cytokine production, as noted in Fig. 11. MIA PaCa-2 does not show sensitivity to IFN-λ1–3 although clearly produces IFN-λs (Fig. 9 and 13). Additionally, we cannot rule out that alternative PDAC cell lines not displaying a heightened antiviral response after METTL3 downregulation, such as Capan-1, may be sensitive to IFNs but do not obtain the ability to produce IFNs. It is well established that different tissue types utilize distinct interferon type signaling pathways (Type I, II, or III) to coordinate immune responses, with each type tailored to specific cellular environments and functions (88). Type III IFNs have been known to be prevalent in the gastrointestinal and respiratory tracts (88, 89) while type I/II IFNs have not been identified as restricted to tissue types but crucial in infiltration of immune cells (90). The induction of cytokines in A549 certainly exemplifies the diversity and abundance of cytokine production in cancer cell lines alternative to PDAC (Fig. 14). In the future, it would be interesting to investigate these conditions for other cancers.

Fundamental hallmarks of cancer include the ability to avoid apoptosis and maintain an indefinite proliferative state (76). It has been found and now widely accepted that m6A regulators play a crucial role in the initiation and maintenance of proliferation in addition to playing an anti-apoptosis role, therefore, contributing to the development and progression of multiple malignancies (91–94). Specifically, the upregulation of METTL3 is involved in the onset, progression, chemo-resistance, immune evasion, and poor prognosis of PDAC by modulating m6A modifications on mRNAs of key oncogenes and tumor suppressor genes (22). We demonstrate here that the cellular response to downregulated METTL3 dramatically upregulates ISGs, which may stunt cancer cell proliferation and contribute to apoptosis. We propose that, for this reason, it may be favorable for cancer cells to upregulate genes, such as METTL3, that aid in evading cancer immunosurveillance. However, our research indicates that METTL3 expression in cancer cells makes PDACs more permissive to VSV and potentially other oncolytic viruses. Also, targeting METTL3 expression in PDAC cells could offer therapeutic benefits and serve as a valuable biomarker for diagnosis and prognosis. Ongoing research, including clinical trial NCT05584111, is exploring the potential of targeting m6A-associated proteins, particularly METTL3, for therapeutic interventions against advanced cancers (95, 96). However, our study suggests that a combined administration of METTL3 inhibitors and OV therapy should be avoided.

## Data Availability

Raw reads generated from this study and the analyzed count table are available at NCBI under the BioProject PRJNA1152582.

## SUPPLEMENTAL MATERIAL

Figure S1 jvi.02284-24-s0001.eps Gene ontology terms that are significantly enriched in siMETTL3-treated SUIT-2 compared to siSCR-treated SUIT-2.

Figure S2 jvi.02284-24-s0002.eps Gene ontology terms that are significantly enriched in siMETTL3-treated SUIT-2 compared to siSCR-treated MIA PaCa-2.

Figure S3 jvi.02284-24-s0003.eps Differentially expressed genes and pathways in PDAC cell lines after siRNA treatment. 

Figure S4 jvi.02284-24-s0004.eps Ingenuity Pathway Analysis (IPA) illustrating “Regulation of innate immune response by RNA sensing molecules” pathway gene products enriched in siSCR and siMETTL3 treated PDAC cells.

Figure S5 jvi.02284-24-s0005.eps Ingenuity Pathway Analysis (IPA) illustrating “Interferon signaling” pathway gene products enriched in siSCR and siMETTL3 treated PDAC cells.

ASM does not own the copyrights to Supplemental Material that may be linked to, or accessed through, an article. The authors have granted ASM a non-exclusive, world-wide license to publish the Supplemental Material files. Please contact the corresponding author directly for reuse.
